# Heat inducible nuclear translocation of Kdm6bb drives temperature dependent sex reversal in Nile tilapia

**DOI:** 10.1371/journal.pgen.1011664

**Published:** 2025-04-30

**Authors:** Jigang Lu, Siqi Huang, Shicen Wei, Jiangbo Cheng, Wei Li, Yueyue Fei, Jihui Yang, Ruiqin Hu, Songqian Huang, Wanying Zhai, Zhichao Wu, Mingli Liu, Qianghua Xu, Peng Hu, Liangbiao Chen

**Affiliations:** 1 Key Laboratory of Exploration and Utilization of Aquatic Genetic Resources, Ministry of Education, Shanghai Ocean University, Shanghai, China; 2 International Research Center for Marine Biosciences, Ministry of Science and Technology, Shanghai Ocean University, Shanghai, China; 3 College of Fisheries and Life Science, Shanghai Ocean University, Shanghai, China; 4 The State Key Laboratory of Grassland Agro-ecosystems, College of Pastoral Agriculture Science and Technology, Lanzhou University, Lanzhou, Gansu, China; ZWU: Zhejiang Wanli University, CHINA

## Abstract

Understanding the primary molecular events driving temperature-dependent sex reversal (TSR) has proven challenging, particularly in distinguishing these from secondary effects of sexual differentiation. The mechanisms translating temperature into a sex-determining signal in fish are still largely unknown. Through combined transcriptomic and genome-wide histone methylation analyses of gonads in Nile tilapia (*Oreochromis niloticus*) exposed to normal and elevated temperatures, we observed significant upregulation of male-promoting genes (*amh*, *dmrt1*, *gsdf*) and suppression of female-promoting genes (*wt1a* and *foxl3*) at high temperature. These changes were correlated with methylation changes in H3K27 and H3K4 in the promoter regions of these genes. Among the histone methylation enzymes induced by high temperature, we identified the H3K27 demethylase Kdm6bb to be a key factor. Gene deletion and biochemical studies confirmed that Kdm6bb significantly impacts the H3K27 methylation level, that influences sex determination. Crucially, we discovered that the TSR function of Kdm6bb is mediated by the alternative inclusion of a previously unrecognized intron, enabling nuclear translocation of the demethylase to perform its function. Our findings refute the previously proposed “translation deficiency” mechanism of *kdm6bb*, and highlight the critical role of mRNA alternative splicing and subcellular localization of the demethylase in temperature-induced sex reversal.

## Summary

Temperature-dependent sex reversal (TSR) is a fascinating phenomenon in which environmental temperature influences sex determination, but the underlying molecular mechanisms remain poorly understood. In this study, we explore how high temperature induces masculinization in Nile tilapia by focusing on the role of the H3K27 demethylase Kdm6bb. Our findings uncover a novel mechanism where the inclusion of an intron (intron 8) in the kdm6bb transcript facilitates its nuclear translocation. Once inside the nucleus, Kdm6bb demethylates H3K27, triggering a cascade of genetic events that lead to male sex determination. Using experimental validation with CRISPR/Cas9 knockouts and overexpression studies, we demonstrate that the alternative splicing of kdm6bb is crucial for its proper subcellular localization and function in temperature-induced sex reversal. Our research emphasizes the importance of alternative splicing and epigenetic regulation in sex determination, providing new insights into environmentally sensitive mechanisms of sex reversal. This study contributes to our understanding of how genetic and environmental factors interact in sex determination, with broad implications for evolutionary biology and aquaculture, particularly in species affected by climate change.

## Introduction

The variability of sex-determining mechanisms in lower vertebrates poses a significant paradox in developmental biology [1]. While mammals and birds rely on a chromosomal sex determination (CSD) system, characterized by specific master sex-determining genes (*sry* in mammals, *dmrt1* in birds), fish exhibit a more plastic approach, influenced by genetic and environmental factors [1]. In fish, temperature-dependent sex determination (TSD) is common and provides unique insights into the evolution and regulation of sex determination mechanisms [2,3].

Research across various teleost species, such as African catfish (*Clarias gariepinus*) [4], European sea bass (*Dicentrarchus labrax*) [5], and Japanese flounder (*Paralichthys olivaceus*) [6], has shown that heat exposure during early development upregulates testis differentiation genes and downregulates ovarian differentiation genes, increasing the proportion of phenotypic males [5,7–10]. However, the molecular mechanisms driving heat-induced sex reversal in fish remain largely unclear.

Epigenetic regulation plays a crucial role in environmental sex determination in reptiles and sex reversal in mammals [11–13]. Modifications such as histone and DNA methylation affect gene expression by influencing transcription factor binding and chromatin remodeling [14–16]. Furthermore, lysine methylation can occur in different forms (mono-, di-, or trimethylation) and is mediated by lysine methyltransferases and demethylases that target both histone and non-histone proteins [17]. Among histone demethylases, Kdm6b has been identified as a key player in sex determination in reptiles [12]. In Nile tilapia, recent studies have implicated the paralog Kdm6bb in high temperature-induced sex reversal (TSR), but the proposed mechanism involving a translationally defective variant due to intron retention lacked molecular support [18].

Nile tilapia, with its well-characterized XY sex determination system and susceptibility to temperature-induced sex reversal, serves as an excellent model for exploring TSR mechanisms [19,20]. High-temperature treatment shortly after fertilization can induce genetic XX females to develop as phenotypic males [21,22]. The availability of genomic data and established sex determination models in this species facilitates detailed molecular studies [23].

In this study, we analyzed transcriptional and chromatin dynamics in Nile tilapia gonads to identify the primary triggers and molecular cascades involved in high temperature-induced masculinization. CRISPR/Cas9 knockouts demonstrated that Kdm6bb plays a critical role in converting genetic females to phenotypic males under high temperature. We identified a temperature-induced alternatively spliced variant of Kdm6bb, including a previously unreported intron (I8), essential for nuclear translocation and subsequent TSR. Our results redefine the understanding of Kdm6bb’s role in TSR, emphasizing the importance of alternative splicing and subcellular localization in sex determination.

## Results

### High temperature induces conversion of genetic females to pseudomales in Nile Tilapia

**Fig 1:**
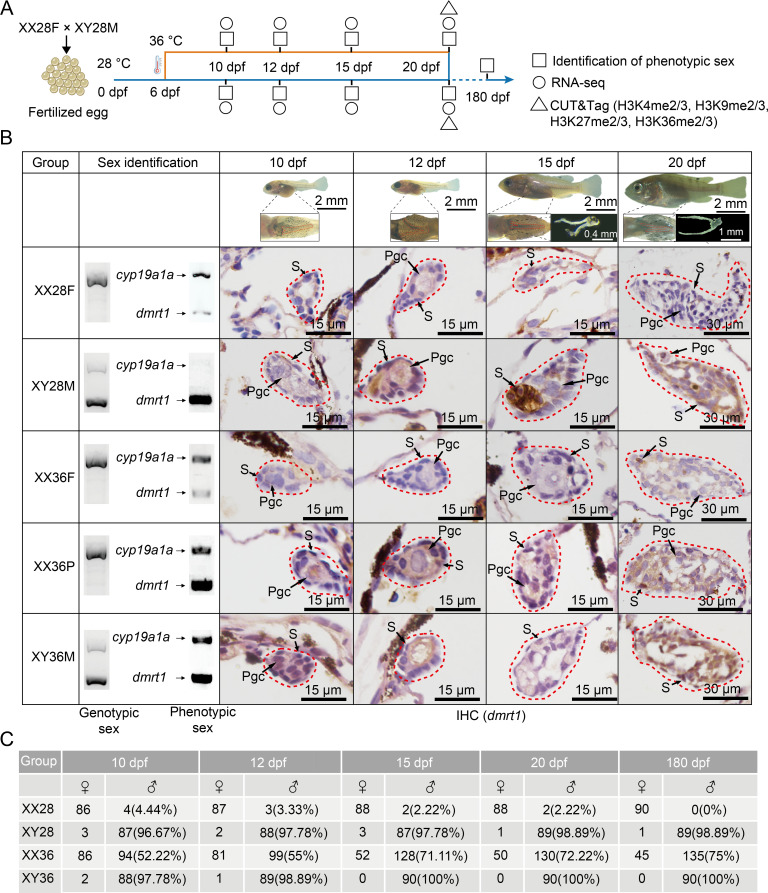
Experimental design and molecular identification for high temperature-induced masculinization. (A) Sampling plan: Nile tilapia larvae were exposed to 36 °C for 15 days or maintained at 28 °C. Gonadal samples were collected at 10, 12, 15, and 20 dpf for transcriptome sequencing and CUT&Tag analysis. (B) Sampling stages: Images depict tilapia at various stages and gonad appearance at 15 and 20 dpf (Top). Genetic and phenotypic typing: Gel images and immunohistochemical staining for DMRT1 in gonads. “XX28F” refers to genetic XX females at 28 °C, while “XX36P” refers to genetic XX pseudomales at 36 °C, the others follow the same rule of nomenclature. PCR with sex-specific markers and RT-PCR with *dmrt1* and *cyp19a1a* primers were used for genotypic and phenotypic sex determination, respectively (Bottom). Pgc, Primordial germ cell; S, gonadal somatic cell. (C) Phenotypic typing statistics: Phenotypic sex typing statistics for the XX28, XY28, XX36, and XY36 groups at various developmental stages using RT-PCR quantification of *dmrt1* and *cyp19a1a* at 10-20 dpf and visual examination at 180 dpf.

Thousands of tilapia larvae produced by wildtype XX (female) and XY (male) parents were raised at a normal temperature (28 °C) until 6 days post-fertilization (dpf). At this stage, the larvae, while still carrying a yolk sac, exhibited free-swimming capability. Furthermore, gonadal primordia were not yet formed by this time, with development beginning between 8 and 10 dpf [24]. They were then divided into two groups: one subjected to the normal 28 °C treatment and the other to a sex reversal temperature of 36 °C until 20 dpf, after which both groups were returned to 28 °C until sex maturation (Fig 1A). We collected gonadal samples at 10, 12, 15, and 20 dpf from both groups. The relative expression levels of *dmrt1* and *cyp19a1a*, along with genotyping using sex-specific primers, were used to assess the phenotypic and genetic sex of each larva (S1 Data) [25]. Based on these measures, samples were categorized into five temperature-sex groups: XX28F (female at 28 °C), XY28M (male at 28 °C), XX36F (genetic female remaining female after 36 °C treatment), XX36P (genetic female converted to pseudomale after 36 °C treatment), and XY36M (genetic male after 36 °C treatment) (Fig 1B and 1C).

The results of immunohistochemical staining (IHC) showed that the Dmrt1 was predominantly expressed in the cytoplasm and nucleus of gonadal somatic cells. In addition, the level of Dmrt1 increased strongly in phenotypic male gonads compared to phenotypic females. By 20 dpf, the Dmrt1 level in XX36P exceeded that in XX28F and matched that in XY28M, indicating successful sex reversal (Fig 1B). The ratio of sex reversal from XX female to pseudomale under this temperature treatment scheme was 75%, as assessed from sexually mature individuals at 180 dpf. No XY males reversed to pseudofemales. This ratio was consistent with assessments using the relative expression of *dmrt1* and *cyp19a1a* at 20 dpf, confirming the reliability of these markers for sex identification in Nile tilapia (Fig 1C).

### Transcriptional changes of sex-biased genes during high temperature-induced sex reversal

**Fig 2:**
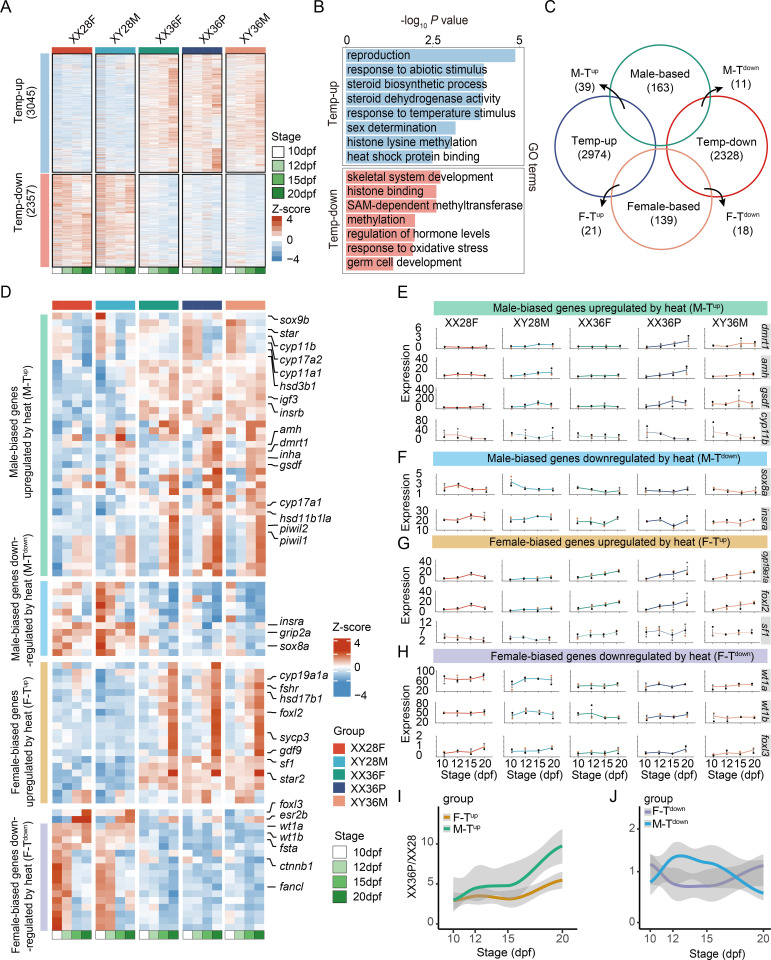
Transcriptional atlas of high temperature-induced masculinization in Nile tilapia. (A) Gene clusters heatmap: Heatmap showing altered gene expression in Temp-up and Temp-down clusters. (B) GO enrichment analysis: Histogram summarizing GO term functions for Differentially expressed genes (DEGs) between 36 °C and 28 °C samples using REViGO. (C) Euler diagram: Number of transcripts uniquely assigned to each region. (D) Gene clusters heatmap: Altered expression in four clusters: Male-biased genes upregulated by heat (M-T^up^); Male-biased genes downregulated by heat (M-T^down^); Female-biased genes upregulated by heat (F-T^up^); Female-biased genes downregulated by heat (F-T^down^). (E-H) Expression patterns: Expression of representative genes in genetic-phenotypic sex groups across developmental stages. The data points derived from the same individual are denoted by the same color. (I-J) The overall gene expression patterns were analyzed by comparing the ratio of the expression of XX36P to those of XX28F (XX36P/XX28F) in the M-T^up^, M-T^down^, F-T^up^, and F-T^down^ clusters along the developmental stages (sampled stages).

RNA sequencing of all time-series samples was conducted to capture transcriptional changes during the temperature treatment. An average alignment and filtering retention rate of 90.16% was achieved across samples (S2 Data). Significant transcriptional variations were observed among the five groups of gonads, largely clustering by developmental stages (S1A Fig). Differentially expressed genes (DEGs) were identified using a threshold of *P* < 0.05 and fold change > 1. *K-means* clustering analysis identified 3034 high-temperature-upregulated genes (Temp-up) and 2357 high-temperature-downregulated genes (Temp-down) (Fig 2A and S3 Data). Additionally, 213 male-biased and 179 female-biased genes were identified from the XX28F and XY28M groups (S1B Fig and S4 Data). Gene Ontology (GO) analysis of the DEGs revealed significant enrichment in terms related to germ cell development, sex determination, reproduction, and histone lysine methylation (Fig 2B and S5 Data).

DEGs between XX28F and XY28M that were also temperature-sensitive were categorized into four clusters: male-biased genes upregulated by heat (M-T^up^), male-biased genes downregulated by heat (M-T^down^), female-biased genes upregulated by heat (F-T^up^), and female-biased genes downregulated by heat (F-T^down^) (Fig 2C and S6 Data). M-T^up^ included classical testis-specific genes such as *dmrt1*, *amh*, *gsdf*, and *sox9b* (Figs 2D and 2E). This cluster also contained genes involved in hormone synthesis, consistent with previous findings on environmental sex determination [26–28]. In contrast, M-T^down^ included genes like *grip2a*, *sox8a*, and *insra* (Fig 2D and 2F). Responses of female-biased genes to high temperature were more complex, with some upregulated (e.g., *cyp19a1a*, *foxl2*, *sf1*, *fshr*) and others downregulated (e.g., *foxl3*, *wt1a*, *wt1b*, *fsta*, *ctnnb1*) (Fig 2D, 2G, and 2H).

The ratio of gene expression in XX36P to XX28F (XX36P/XX28F) indicated the extent of gene expression changes in high temperature-induced pseudomales compared to normal XX females. The XX36P/XX28F ratio for M-T^up^ and F-T^up^ showed an increasing trend from 10 to 20 dpf, with M-T^up^ significantly higher than F-T^up^ from 15 dpf onwards (Fig 2I). Conversely, the XX36P/XX28F ratio in M-T^down^ initially rose then fell, while in F-T^down^ it first decreased then increased, resulting in a lower ratio in M-T^down^ compared to F-T^down^ at 20 dpf (Fig 2J). Overall, the gene expression data from our temperature-induced sex reversal were consistent with factors known to influence genetic and temperature-dependent sex determination in fish and mice (S1C Fig).

### High temperature-induced masculinization involves extensive epigenetic reprogramming

To elucidate the epigenetic modifications underlying transcriptional changes in sex-biased genes during TSR, we conducted Cleavage Under Targets and Tagmentation (CUT&Tag) on 20 dpf gonadal samples. Di- and tri-methylation profiles at four histone positions (H3K4, H3K9, H3K27, H3K36) were analyzed (S2A Fig), identifying peaks for H3K4me2, H3K4me3, H3K9me2, H3K9me3, H3K27me2, H3K27me3, H3K36me2, and H3K36me3, covering 1.87%, 1.22%, 0.21%, 0.11%, 0.14%, 0.17%, 0.09%, and 0.05% of the genome, respectively (S2B Fig). PCA analysis showed clustering of samples based on histone modification types (S2C Fig). Histone marks associated with transcriptional activation (H3K4me2/3, H3K36me2/3) showed positive correlations with each other and inverse correlations with repressive marks (H3K9me2/3, H3K27me2/3) (S2C Fig). Notably, H3K4me2/3 peaks were predominantly located in promoter regions, unlike other histone modification peaks found mainly in intergenic regions and gene bodies (S2D and S2E Fig).

**Fig 3:**
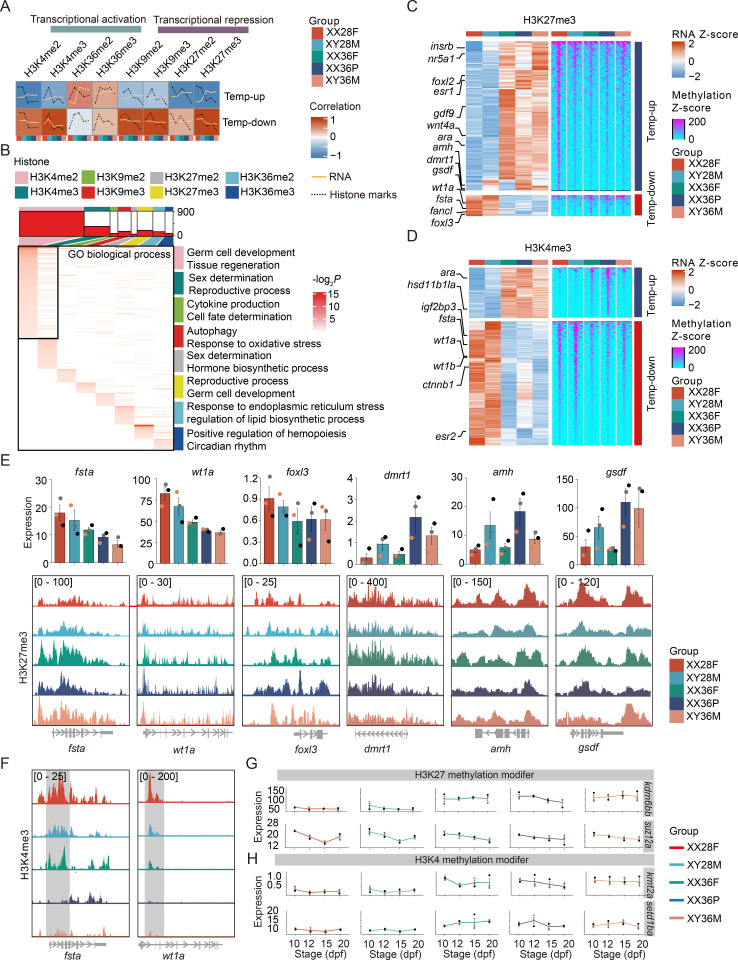
Chromatin dynamics coordinate transcription profiling during high temperature-induced masculinization. (A) Correlation analysis between transcriptional changes and types of epigenetic marks at 20 dpf for high temperature induced (Temp-up) and high temperature repressed (Temp-down) gene clusters. In each cluster, the solid line represents gene expression levels, while the dashed line represents the level of the corresponding histone methylation mark. The vertical axis indicates the Z-score for genes or peaks within that cluster, and the horizontal axis represents the temperature-sex groups (i.e., XX28F, XY28M, XX36F, XX36P, XY36M). (B) Number of peaks of the 8 histone methylation marks and the GO biological process terms represented by the genes associated with the peaks. The top section (columns) displays the number of peaks for each histone methylation type, with colors representing each type of histone modification as indicated by the legend. The bottom section (heatmap) shows the functional enrichment of proximal genes for each histone methylation type based on GO biological processes. Columns represent the 8 histone methylation types, and rows represent GO terms within each type. All GO terms are presented in S9 Data. Heatmap notes summarize the functions of nearby GO terms using REViGO. (C - D) Heatmap displaying transcriptional dynamics and the level of modifications of H3K27me3 (C) and H3K4me3 (D) for the temperature responsive genes, with PCC below -0.5 for H3K27me3 and above 0.5 for H3K4me3. The left side lists the representative genes known to be involved in sex determination. (E) Dynamic transcription (top) and H3K27me3 modification tracks (bottom) for three representative female-biased genes (*fata*, *wt1a*, and *foxl3*) and three male-biased genes (*dmrt1*, *amh*, and *gsdf*). Gene expression data are shown as mean ± SEM of 3 biological replicates. The data points derived from the same individual are denoted by the same color. (F) H3K4me3 modification tracks for *fsta* and *wt1a*. (G) The dynamic expression of *kdm6bb* and *jarid2b*, which function in H3K27 methyl-modification along the developmental stages. The data points derived from the same individual are denoted by the same color. (H) The dynamic expression of *kmt2a* and *setd1ba,* which function in H3K4 methyl-modification along the developmental stages. The data points derived from the same individual are denoted by the same color.

We assessed the Pearson correlation coefficients (PCCs) between transcriptional patterns (Temp-up and Temp-down) and histone modifications (Fig 3A and S7 Data). Most histone marks, including H3K4me2/3, H3K9me2/3, and H3K27me2/3, showed a negative correlation with transcriptional changes in the Temp-up clusters, particularly H3K27me2/3. Almost all histone marks, except H3K36me2, showed a positive correlation with transcriptional changes in the Temp-down cluster, particularly H3K4me3 (Fig 3A). We identified genes with significant correlations between histone methylation and transcriptional expression, revealing distinct biological functions associated with these epigenetic modifications (Fig 3B and S8 and S9 Data files). For instance, H3K4me2/3 were associated with many developmental functions such as germ cell development and reproductive processes (Fig 3B). H3K27me2 was expectedly involved in sex determination and hormone biosynthesis, and H3K27me3 in reproduction and germ cell development (Fig 3B).

Genes involved in male determination (e.g., *dmrt1*, *amh*, *gsdf*) and female differentiation (e.g., *foxl3*, *fsta*, *wt1a*, *fancl*) were strongly negatively correlated with H3K27me3 and H3K27me2 levels (Figs 3C, 3E, S3A, and S3C). Some female-biased genes (*wt1a*, *wt1b*, *ctnnb1*) also showed positive correlations with H3K4me3 and H3K4me2 levels (Figs 3D, 3F, S3B, and S3D). Among the 38 temperature-influenced epigenetic modifiers identified, H3K4 modifiers (e.g., *kmt2a*, *kmt2d*, *setd1ba*) and H3K27 modifiers (e.g., *kdm6bb*, *jarid2b*) were significantly upregulated by high temperature (Figs 3G, 3H, S4B, and S4D). Given its strong induction and significant negative correlation with H3K27 methylation, we further investigated the role of *kdm6bb* in TSR in Nile tilapia.

### Reduced Kdm6bb triggers male-to-female conversion in XY tilapia

**Fig 4:**
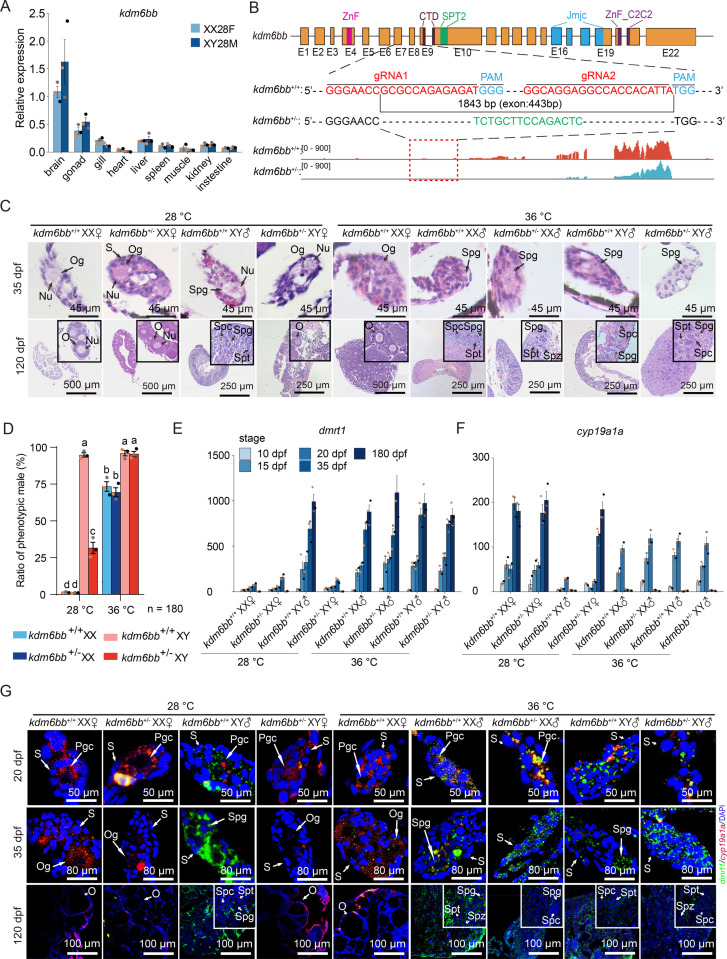
Loss of *kdm6bb* leads to male-to-female sex reversal in XY Nile tilapia raised in 28 °C. (A) qRT-PCR analysis of *kdm6bb* expression in various tissues at 20 dpf. The data points derived from the same individual are denoted by the same color. (B) Schematic representation of gRNA targeting the *kdm6bb* gene, showing the deletion of an 1843 bp fragment, including 443 bp of exonic sequence spanning from Exons 7 to 9, and the insertion of 15 base pairs (highlighted in green). Below is a screenshot of *kdm6bb* RNA-Seq data in tilapia gonads from the IGV browser. (C) Histology sectioning and H&E staining of tilapia gonads at 35 and 120 dpf. Nu, nucleoli; Og, oogonia; O, oocyte; Spg, spermatogonia; Spc, spermatocyte; Spt, spermatid; Spz, spermatozoa. (D) Ratio of phenotypic males developed in various *kdm6bb* genotypes of Nile tilapia, with and without high-temperature treatment. Gonadal sex was determined by morphological analysis of gonads from four-month-old fish using H&E staining. Data were derived from three independent experiments, each with 60 fish per group. The data points derived from the same individual are denoted by the same color. (E-F) qRT-PCR analysis of *dmrt1* and *cyp19a1a* expression in different *kdm6bb* genotypes. The data points derived from the same individual are denoted by the same color. (G) Fluorescence *in situ* hybridization (FISH) of *dmrt1* and *cyp19a1a* in gonadal sections of tilapia at 25, 35, and 120 dpf, indicating relative mRNA levels of *dmrt1* and *cyp19a1a* in different *kdm6bb* genotypes and under different temperature treatments (28 °C or 36 °C). DAPI staining was used to visualize nuclei. Pgc, Primordial germ cell; Og, oogonia; O, oocyte; Spg, spermatogonia; Spc, spermatocyte; Spt, spermatid; Spz, spermatozoa; S, gonadal somatic cell.

Quantitative analysis of *kdm6bb* mRNA in various tissues of 20 dpf tilapia at 28 °C revealed predominant expression in the brain and gonads, with little sexual dimorphism (Fig 4A). To investigate *kdm6bb*’s role in high temperature-induced masculinization, we created a *kdm6bb* knockout model using CRISPR/Cas9, deleting an 1843 bp fragment from exons 7–9, causing a frameshift and premature stop codons (Figs 4B, S5A, and S5B). Heterozygous deletion reduced *kdm6bb* mRNA levels, while homozygotes exhibited high lethality, with survival rates of 2.65% at 5 dpf and about 1% at 180 dpf (S5C Fig).

We examined gonadal development in *kdm6bb*^*+/-*^ mutants and *kdm6bb*^+/+^ siblings subjected to high temperature (36 °C) treatment as per Fig 1A, using H&E staining at 35 and 120 dpf. At 28 °C, both *kdm6bb*^*+/+*^ and *kdm6bb*^*+/-*^ XX tilapia developed oogonia by 35 dpf and matured into oocytes by 120 dpf (Fig 4C). However, only 32.7% of *kdm6bb*^*+/-*^ XY gonads developed spermatogonia and differentiated into spermatozoa by 120 dpf, with a 67.3% reduction in males (Fig 4D). Despite *kdm6bb* reduction, *kdm6bb*^*+/-*^ tilapia responded to high temperature, with 72–75% of XX fish developing into pseudomales, similar to *kdm6bb*^+/+^ (Fig 4D). This shows that high temperature can induce sex reversal even with reduced *kdm6bb*.

We traced the expression of three male-biased genes (*dmrt1, gsdf, amh*) and two female-biased genes (*foxl2, cyp19a1a*) at multiple stages (10, 15, 20, 35, 120 dpf). These genes showed slow mRNA accumulation initially, with dramatic changes at 35 and 120 dpf (Figs 4E, 4F, and S5D). Fluorescence *In situ* hybridization (FISH) confirmed *dmrt1* expression in male gonads and *cyp19a1a* in female gonads at 20, 35, and 120 dpf (Fig 4G). Reduced *kdm6bb* in *kdm6bb*^+/-^ XY fish increased pseudofemales, with higher female-biased gene expression and lower male-specific gene expression (Fig 4E, 4F, and 4G). These results establish *kdm6bb*’s role in sex determination in Nile tilapia.

### High temperature-induced nuclear translocation of Kdm6bb

**Fig 5:**
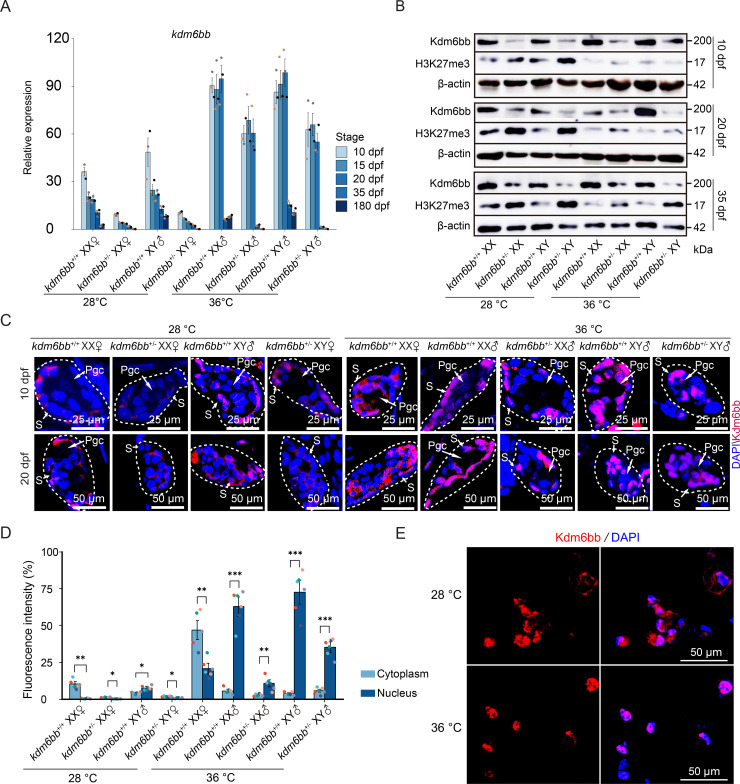
High temperature-induced H3K27me3 demethylation activity and nuclear translocation of Kdm6bb in Nile tilapia. (A) Expression levels of *kdm6bb* in *kdm6bb*^+/+^ and *kdm6bb*^+/-^ individuals under normal and high-temperature conditions in the gonads. The data points derived from the same individual are denoted by the same color. (B) Western blot analysis of Kdm6bb and H3K27me3 expression in tilapia gonads at 10, 20, and 35 dpf, demonstrating the negative correlation between H3K27me3 levels and Kdm6bb expression. (C) Immunofluorescence staining of Kdm6bb (red) in gonadal sections of 10 and 20 dpf at 28 °C and 36 °C from *kdm6bb*^+/+^ and *kdm6bb*^+/-^ individuals. Dotted circles outline the gonadal areas. DAPI (blue) was used to stain nuclei. Pgc, Primordial germ cell; S, gonadal somatic cell. (D) The relative abundance of Kdm6bb protein located in the nucleus and cytoplasm. The fluorescence intensity located to nuclei or cytoplasm of each cell was normalized to the total areas of nuclei (represented by DAPI staining area) and the average ratio (nuclei/cytoplasm) of 20 cells were calculated. (*P < 0.05, **P < 0.01, ***P < 0.001). The data points derived from the same individual are denoted by the same color. (E) Enhanced nuclear translocation of Kdm6bb in a tilapia brain cell line incubated at 36 °C, as shown by immunohistochemical staining using Kdm6bb antibody.

To explore the functionality of Kdm6bb in temperature-dependent sex reversal (TSR), we first quantified its mRNA levels. In 20 dpf tilapia raised at 28 °C, *kdm6bb* mRNA levels were low in gonads and further reduced in *kdm6bb*^*+/-*^ individuals. When exposed to high temperature, *kdm6bb* mRNA levels increased 3–5 folds, but quickly returned to basal levels when the temperature normalized at 35 dpf, indicating a strong temperature inducibility of this gene (Fig 5A).

We performed Western blot analyses for Kdm6bb and H3K27me3 in gonads at 10, 20, and 35 dpf. Kdm6bb expression was consistently lower in heterozygotes than in *kdm6bb*^+/+^ at all stages and conditions (Fig 5B). Significant increases in Kdm6bb protein levels were observed in *kdm6bb*^+/+^ samples at 10 and 20 dpf under 36 °C treatment. Notably, no significant reduction in Kdm6bb was seen at 35 dpf when *kdm6bb* mRNA had returned to basal levels (Fig 5A and 5B). There was a clear negative correlation between Kdm6bb and H3K27me3 levels across all samples, suggesting that Kdm6bb directly demethylates H3K27 in the gonads. Specifically, H3K27me3 levels were lower in 36 °C-treated gonads compared to 28 °C samples, particularly at 10 and 20 dpf (Fig 5B). These findings establish Kdm6bb as a key regulator of H3K27me3 and sex-specific gene expression during sex determination.

To further investigate Kdm6bb’s role in sex determination, we conducted immunofluorescence staining of Kdm6bb in gonads collected at 10 and 20 dpf. Immunofluorescence showed that protein of Kdm6bb were detected in gonadal somatic cells but not germ cells (Fig 5C), implying that Kdm6bb functions in gonadal somatic cells to regulate the sexual development of Nile tilapia. This analysis also confirmed a significant reduction in Kdm6bb in the gonadal cells of *kdm6bb*^*+/-*^ individuals compared to *kdm6bb*^+/+^ at both time points (Fig 5C). Elevated temperatures increased Kdm6bb protein levels relative to normal (28 °C) conditions. Most notably, we observed distinct subcellular localizations of Kdm6bb: it was primarily cytoplasmic in phenotypic females, while predominantly nuclear in phenotypic males, especially at 36 °C (Fig 5C). Statistical analysis of gonads from three fish per group (6 gonads total) demonstrated significantly higher proportions of nuclear-localized Kdm6bb in phenotypic males compared to *kdm6bb*^*+/+*^ XX females and *kdm6bb*^*+/-*^ XY pseudofemales (Fig 5D).

To further validate the increased nuclear localization of Kdm6bb at high temperature, we cultured TBN cells, a cell line derived from tilapia brain, where *kdm6bb* is abundantly expressed (Fig 4A). Kdm6bb was distributed in both the nucleus and cytoplasm of TBN cells cultured at 28 °C but localized exclusively to the nuclei of cells cultured at 36 °C (Fig 5E). These results clearly demonstrate that high temperature induces the nuclear translocation of Kdm6bb.

### Identification of Kdm6bb variants responsible for high temperature-induced nuclear translocation and sex reversal

**Fig 6:**
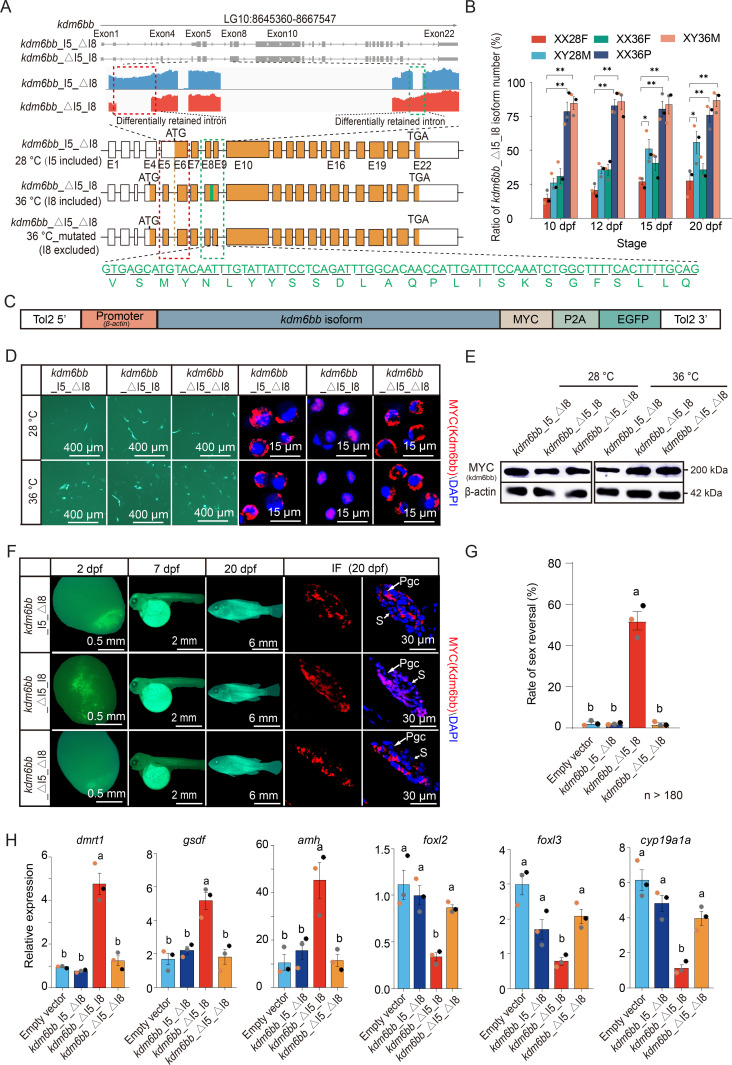
Identification of the *kdm6bb* transcript variant conferring high temperature-induced sex reversal. (A) Top: Screenshots of *kdm6bb* RNA-Seq data in tilapia gonads displayed using IGV browser. Bottom: Exon-intron arrangements of the predominant splicing variants at 28 °C (*kdm6bb*_I5_△I8) and 36 °C (*kdm6bb*_△I5_I8), along with the 36 °C-mutated variant (*kdm6bb*_△I5_△I8) constructed to test the function of I8. Regions with alternative splicing are boxed with dotted lines, and the translated amino acids of I8 are shown in green. “ATG” denotes the start codon for translation, and “TGA” denotes the stop codon. (B) Relative percentage of the *kdm6bb*_△I5_I8 isoform compared to the total count of *kdm6bb* transcripts in gonads at 10, 12, 15, and 20 dpf stages for each genotype-phenotype sex combination. The data points derived from the same individual are denoted by the same color. (C) Expression construct for the *kdm6bb* isoforms. Promoter, *β-actin* promoter, MYC，the protein tag fused to Kdm6bb isoform; P2A, the protein cleavage signal peptide. (D) Transfection of the *kdm6bb* constructs into the tilapia brain cell line (TBN) cultured at 28 °C and 36 °C. Immunohistochemical staining of the Kdm6bb-MYC fusion protein using MYC antibody shows the relative subcellular localization (nuclei or cytoplasm) of the protein product expressed by the three constructs. (E) Western blot analysis showed the expression of the MYC-tagged plasmids fusion protein in Nile tilapia gonads raised at 28 °C and 36 °C after transfection of the three constructs into one-cell embryos. (F) Examination of EGFP expression and Kdm6bb-MYC in the entire fish, along with immunohistochemical staining of MYC in gonadal sections, indicate the nuclear localization of the *kdm6bb*_△I5_I8 product in transgenic fish raised at 28 °C. Pgc, Primordial germ cell; S, gonadal somatic cell. (G) Ratio of sex reversal achieved in transgenic fish overexpressing one of the three *kdm6bb* isoforms at 28 °C. Gonadal sex was determined by RT-PCR analysis of *dmrt1* and *cyp19a1a* expression levels in gonads at 20 dpf. The data points derived from the same individual are denoted by the same color. (H) Relative expression of male-biased genes (*dmrt1*, *gsdf*, and *amh*) and female-biased genes (*foxl2*, *foxl3*, and *cyp19a1a*) in transgenic fish expressing one of the three *kdm6bb* constructs raised at 28 °C. The data points derived from the same individual are denoted by the same color.

Analysis of *kdm6bb* transcripts from samples treated at 28 °C and 36 °C identified two distinct variants: *kdm6bb_*I5_△I8 and *kdm6bb_*△I5_I8 in the gonads and brains of tilapia (Figs 6A and S6A). These variants differ by the alternative splicing of introns I5 and I8. RNA-seq data from XX28F, XY28M, XX36F, XX36P, and XY36M gonads showed that high temperature significantly increased the proportion of *kdm6bb_*△I5_I8 in XX36P and XY36M gonads, which developed into phenotypic males. Higher rates of *kdm6bb_*△I5_I8 compared to *kdm6bb_*I5_△I8 were also observed in XY28M individuals. Conversely, phenotypic females (XX28F, XX36F) had significantly lower amounts of *kdm6bb_*△I5_I8 (Figs 6B and S6C). Additionally, a small fraction (about 7%) of the *kdm6bb*_△I5_△I8 isoform in which both introns are absent was also detected under 36 °C (S6B Fig). This result implies that *kdm6bb_*△I5_I8 might be responsible for TSR and promoting male differentiation at normal temperatures.

To determine which variant is responsible for nuclear translocation and TSR, we cloned *kdm6bb_*I5_△I8, *kdm6bb_*△I5_I8, and *kdm6bb_*△I5_△I8 (a mutated variant with the I8 sequence removed) into an expression vector with a MYC tag and EGFP (Fig 6C). Transfecting these constructs into TBN cells and culturing them at 28 °C and 36 °C, we used immunohistochemical staining against MYC to localize the exogenous Kdm6bb variants. The products of *kdm6bb_*I5_△I8 and *kdm6bb_*△I5_△I8 were predominantly cytoplasmic, while *kdm6bb_*△I5_I8 was almost exclusively nuclear (Fig 6D). This pattern was consistent at both temperatures, indicating that the inclusion of I8 is essential and sufficient for nuclear translocation of Kdm6bb. Immunoblotting confirmed equal protein levels from all three variants, refuting the previous assumption that *kdm6bb_*I5_△I8 was translationally defective (Fig 6E).

To validate the in vivo effects, we microinjected the three constructs into fertilized tilapia eggs (Fig 6F). The transgenic eggs were hatched and kept at 28 °C until sex differentiation. Histochemical staining showed that *kdm6bb_*I5_△I8 and *kdm6bb_*△I5_△I8 localized to the cytoplasm, while *kdm6bb_*△I5_I8 localized to the nuclei (Fig 6F). The rate of sex reversal in the *kdm6bb_*△I5_I8 overexpressing XX Tilapia was about 50%, significantly higher than those overexpressing *kdm6bb_*I5_△I8, *kdm6bb_*△I5_△I8, or the empty vector (Figs 6G, S6D, and S6E). Male-biased genes (*dmrt1, gsdf, amh*) were upregulated, and female-biased genes (*foxl3, foxl2, cyp19a1a, fsta*) were downregulated only in *kdm6bb_*△I5_I8 expressing fish (Figs 6H and S6F). No significant changes of these sex-biased genes were detected in fish with the other two constructs.

**Fig 7:**
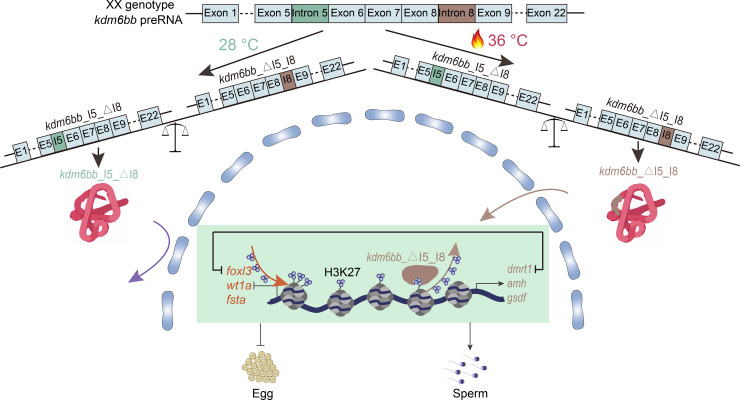
Molecular Mechanism of *kdm6bb* in High Temperature-Induced Masculinization in Tilapia. This diagram illustrates the alternative splicing of *kdm6bb* pre-mRNA under different temperature conditions in XX genotype tilapia. At 28 °C, the predominant splice variant is *kdm6bb_*I5_△I8, which remains cytoplasmic and does not demethylate H3K27, allowing expression of female-biased genes (*foxl3, wt1a, fsta*). At 36 °C, *kdm6bb_*△I5_I8 is the predominant variant, which translocates to the nucleus and demethylates H3K27, promoting male-biased genes (*dmrt1, amh, gsdf*) expression, leading to masculinization. The process results in phenotypic males (sperm) and females (egg) depending on the splicing variant and temperature conditions.

These results indicate that *kdm6bb_*△I5_I8, with its nuclear localization, is the variant capable of causing sex reversal. Nuclear-localized Kdm6bb reduces H3K27 methylation in male-determining gene promoters and suppresses female-biased genes through interactions between *dmrt1* and *foxl2/foxl3* [29–31], shifting the sex determination program of XX genetic females to males. The mechanisms are depicted in Fig 7.

## Discussion

Sex determination traditionally seen in mammals and birds, is more flexible in fish. In mammals, genes like *wt1* and the RSPO1/WNT4/CTNNB1 pathways, along with *fst* (follistatin), trigger ovarian differentiation [32–34]. *Sry*, and *sf1*, inhibit *foxl2*, to allow *sox9* expression, leading to testis differentiation by upregulating male-specific genes like *dmrt1* and *amh* and downregulating *ctnnb1* [32,35]. These master genes (e.g., *sry*, *dmrt1*, *sox9*) are evolutionarily conserved and adaptable across species [36]. In contrast, fish exhibit high plasticity in sex determination, with temperature affecting gene expression. This study revealed high temperature upregulated male-specific genes like *dmrt1*, *amh*, and *gsdf*, while also increasing female-specific genes like *cyp19a1a*, *foxl2*, and *sf1* in Nile tilapia [25]. However, male-biased genes were upregulated to a greater extent than female-biased genes. Notably, female-biased genes, such as *wt1a* (Wilms’ tumor suppressor transcription factor a), *wt1b*, *fsta*, and especially *foxl3* decreased under high temperature. *Foxl3*, identified as a female-determining factor in medaka and tilapia, antagonizes *dmrt1* in germ cells [29,30]. Overall, male-biased genes dominated sex differentiation under high temperature, contributing to masculinization in tilapia and illustrating the complexity of sex differentiation in fish [32,33].

Epigenetic profiling of gonadal samples showed that histone modifications around the promoter regions of sex-biased genes play essential roles in sex determination. Methylation of H3K27 is key for male sex determination, while H3K27 and H3K4 methylation are associated with female-biased genes (*foxl3, wt1a, wt1b, fsta*). Under normal temperatures, male-determining genes show higher H3K27 methylation, while female-biased genes exhibit higher H3K4 and lower H3K27 methylation. High temperature disrupts these patterns, reducing H3K27 methylation in male-determining genes and H3K4 methylation in female-promoting genes, increasing the male ratio in Nile tilapia. In the *kdm6bb*^+/-^ XY group, the male-to-female ratio decreased by 67.3% (Fig 4D). sex reversal in Nile tilapia is mainly due to homozygous mutations in sex-determining genes, such as *amhy* [37], *dmrt1* [29], *foxl2* [38], and *foxl3* [29], but *kdm6bb* heterozygous XY individuals also experience sex reversal. *Kdm6bb* expression is significantly downregulated in heterozygous individuals (Fig 5A and 5B), disrupting sex differentiation and causing sex reversal in some cases. *Kdm6bb* regulates H3K27 methylation, one of the most important methylation marks in biological systems [39], influencing genes critical for sex differentiation. Overexpression of *kdm6bb* upregulates male-specific genes (e.g., *amh*, *dmrt1*) and downregulate female-specific genes (e.g., *wt1a*, *foxl3*) (Fig 6H). This dual regulation by *kdm6bb* explains why sex reversal can occur in heterozygous individuals. A similar effect observed in red-eared slider turtles (*Trachemys scripta*), where *kdm6bb* knockdown disrupts masculinization, further supporting its role in sex differentiation [12,40].

This study demonstrates that the H3K27 demethylase Kdm6bb is crucial for high temperature-induced sex reversal (TSR) in Nile tilapia, highlighting a novel mechanism involving Intron 8. Inclusion of Intron 8 in the *kdm6bb* transcript facilitates nuclear localization of Kdm6bb, essential for H3K27 demethylation and initiating sex reversal. This challenges previous reports suggesting Intron 5 causes premature termination translation at normal temperature, leading to insufficient Kdm6bb for male sex differentiation in the genetic female gonads [18]. We showed that the *kdm6bb* transcript containing Intron 5 is translated effectively, disproving the notion that the kdm6bb transcript is defective [18]. Notably, at 36 °C, Kdm6bb’s nuclear localization is linked to reduced H3K27me3, underscoring its role in TSR. Both studies share the similarity that overexpression of the I8-containing *kdm6bb* variant, *kdm6bb_*△I5_I8 (*kdm6bb*_tv1), significantly increased the ratio of male tilapia even at 28 °C. In contrast, transcripts lacking I8 failed to enter the nucleus, leading to low rates of pseudomale conversion. This clearly establishes that nuclear translocation of Kdm6bb, facilitated by Intron 8, is the key factor mediating sex reversal. The key difference between the two studies lies in the explanation of the *kdm6bb* mechanism in sex reversal. The previous study neglected the alternative splicing at Intron 8, and misinterpreted Intron 5 as the key factor driving sex reversal.

The phenomenon of differential subcellular localization of Kdm6 is not limited to fish. Research on mice revealed that Kdm6b is primarily expressed in the cytoplasm of dorsal root ganglia cells and in the nuclei of cells in the spinal dorsal horn [41]. Similarly, alternative splicing of *kdm6a* mRNA controls the subcellular localization of the Kdm6a in human bladder cancer cells and normal epithelia [42]. Thus, kdm6b has evolved alternative splicing and nuclear translocation mechanisms across vertebrate species. In Nile tilapia, this mechanism specifically functions to mediate temperature-induced sex reversal under high-temperature conditions. Additionally, Kdm6bb’s subcellular localization may impact its role in both the nucleus and cytoplasm, where it may also regulate non-histone proteins, such as the retinoblastoma protein [43–45]. The homozygous Kdm6bb knockout in tilapia led to non-survival, indicating its vital function in the cytoplasm, beyond histone demethylation in the nuclei.

The *kdm6bb*_△I5_I8 isoform is primarily expressed in XY28M and XX36P groups, while the *kdm6bb*_I5_△I8 isoform is predominantly found in XX28F and XX36F groups. Kdm6bb is primarily localized in the nuclei of gonadal somatic cells in XY28M and XX36P groups, whereas in the cytoplasm of gonadal somatic cells in XX28F and XX36F groups. Our unpublished single-cell RNA sequencing of the tilapia gonads indicates that *kdm6bb* is primarily expressed in male Sertoli cells and female granulosa cells, suggesting its differential distribution in supporting cells of the male and female gonads. We propose that at normal temperature, the differential expression of *kdm6bb* in the Sertoli and granulosa cells leads to distinct alternative splicing patterns, contributing to male or female sex differentiation respectively. At high temperature, the *kdm6bb*_△I5_I8 transcript increases in both the XX and the XY gonads, and when it accumulates to the pre-existing *kdm6bb*_I5_△I8 in the XX gonads, a female-to-male sex-reversal occurs. The detail distribution of *kdm6bb* transcripts under different temperatures warrants further investigation, by which, the molecular process governing sex determination and sex reversal in tilapia would be further uncovered.

Nuclear translocation of proteins is a complex process regulated by various mechanisms. In addition to classical and non-classical nuclear localization signals (NLS) proteins dimerization through subunit binding can also serve as an NLS [46]. Nuclear transport is further regulated by posttranslational modification (PTM) like phosphorylation and acetylation [47,48]. In this study, although the I8 sequence of *kdm6bb* is not rich in basic amino acids, it is abundant in phosphorylatable serine and tyrosine residues. We propose that I8 may create or expose a non-classical NLS, potentially interacting with other partners or cargo to assist in nuclear import, though this requires further investigation. This highlights a novel regulatory mechanism involving alternative splicing and nuclear translocation, expanding our understanding of epigenetic regulation in sex determination.

Alternative splicing significantly impacts biological functions, including sex determination. The expression level of Kdm6b plays a crucial role in the temperature-dependent sex determination (TSD) of slider turtles, where it is predominantly expressed in the nucleus [12]. In our study, at 28 °C, female Nile tilapia primarily express the *kdm6bb*_I5_△I8 isoform, while males predominantly express *kdm6bb*_△I5_I8 isoform. This suggests sex-specific alternative splicing of *kdm6bb* at normal temperature. At normal temperatures, in genetic female fish, Kdm6bb is predominately cytoplasmic and is not required for the development of female sex, half deletion of kdm6bb will not affect this process. Conversely, reduced expression of *kdm6bb*_△I5_I8 in *kdm6bb*^+/-^ XY tilapia hinders the development of male sex, increasing the female ratio. When the temperature raises higher, the proportion of *kdm6bb*_△I5_I8 is elevated in both sex which drives female-to-male sex reversal. Temperature affects the alternative splicing pattern of *kdm6bb*. Our overexpression experiments demonstrated that introducing *kdm6bb*_△I5_I8 alone at 28 °C induces sex reversal. Based on these findings, we conclude that, unlike in slider turtles, where Kdm6b expression level alone is critical for TSD, both the expression level and alternative splicing of *kdm6bb* play pivotal roles in the male sex development and temperature-dependent sex reversal in Nile tilapia. Similar alternative splicing mechanisms in reptiles, amphibians and other species such as in the Wilms’ tumor suppressor gene (*wt1*) in mice, involve the inclusion or exclusion of amino acids affecting protein localization and sex reversal [11,34]. This suggests a conserved regulatory role across taxa. The regulation of *kdm6bb* splicing by temperature likely involves core spliceosomal components and kinases, which modulate splicing through interactions with RNA transcripts.

Our study also observed dynamic expression in the cortisol pathway, known to mediate high-temperature stress in fishes [49]. Increased expression of enzymes controlling cortisol production suggests cortisol’s involvement in high temperature-induced masculinization, consistent with observations in other vertebrates undergoing natural or temperature-induced sex reversal [11,50].

Despite these findings, reduced *kdm6bb* in some *kdm6bb* heterozygous XY Nile tilapia did not undergo sex reversal under normal temperatures, and high temperature did not convert all XX females into pseudomales. This indicates that other factors may also play a role in TSR. Besides *kdm6bb*, high temperature significantly influenced the expression of other H3K27me3 modifiers, such as *suz12a, pcgf3,* and *eed*, as well as H3K4 modifiers like *kmt2a* and *setd1ba*, both epigenetic markers are shown to be related with expression of the male and female determining genes.

In summary, this study reveals how high temperature trigger masculinization in Nile tilapia through a genetic cascade regulated by the H3K27 demethylase *kdm6bb*. This finding sheds light on the evolution of sex determination mechanisms in vertebrates, emphasizing the importance of alternative splicing and subcellular localization of epigenetic factors in environmentally sensitive sex determination.

## Materials and methods

### Ethics

All fish were maintained and experiments were conducted according to the guidelines of the Committee on Laboratory Animal Care and Use of Shanghai Ocean University under protocol # SHOU-DW-2021–061.

### Fish husbandry and temperature treatments

Juvenile Nile tilapia were obtained from Guangxi Fisheries Research Institute (Nanning, China). The fish were reared in a circulating aerated freshwater system at 28 °C with pellet feed of appropriate size (Tianbang, China) under a 14L:10D light/dark photoperiod for over 6 months until maturity. Breeding pairs of XX and XY Nile tilapia spawned, fertilized, and hatched at 28 °C. Fertilized eggs were collected, counted, and hatched in 200 ml brooders (Ziss, Korea) in 40-liter tanks containing embryo medium with methylene blue and maintained at 28 °C. Three different breeding pairs of normal females (XX) and normal males (XY) Nile tilapia were crossed, and their offspring were used in subsequent experiments. Approximately 3000 larvae from these parents were subjected to temperature treatments at 28 °C and 36 °C from 6 dpf to 20 dpf. After that, they were reared at 28 °C until sexual maturity (Fig 1A). Thermal treatment was performed in 130 L glass tanks with a circulating aerated freshwater system maintained by a 2000 W heater (A-MI Corporation, Korea). Water temperatures in the tanks were measured three times per day to ensure constant conditions throughout the treatment period.

### Sampling procedures for RNA-seq and CUT&Tag

Fish of the desired time points were sacrificed using an overdose of MS-222 (Tricaine methanesulfonate) (Sigma-Aldrich, St Louis, MO, USA), followed by tissue sampling. Firstly, DNA from the fish fin was extracted [51]. Genotype identification of the genetic sex of each fish was conducted by PCR using a pair of primers (XX-XY) targeting the sex-specific region of tilapia [52] following Wang et al. [53]. The genetic sex of each fish was assigned according to the sex-specific DNA banding pattern. Then, the four groups obtained included XX females raised at 28 °C (XX28F), XY males raised at 28 °C (XY28M), XY males raised at 36 (XY36M), and XX individuals raised at 36 °C (XX36) (Fig 1B and 1C).

Ninety fish from each of XX28F, XY28M, and XY36M groups, and 180 fish from the XX36 group were sampled at 10, 12, 15, 20, and 180 dpf. Embryonic gonads containing some peritoneum at 10 and 12 dpf, and pure gonads at 15 and 20 dpf were preserved in TRIzol reagent (Life Technologies, Carlsbad, CA, USA) and total RNA were extracted for phenotypic sex identification via RT-PCR using *dmrt1* and *cyp19a1a* primers (S1 Data), following the protocol by Lu et al. [25]. Phenotypic sex was classified as male if the expression level of *dmrt1* was higher than that of *cyp19a1a*; otherwise, it was classified as phenotypic female. For 180 dpf fish, phenotypic sex was identified by visually observing the urogenital papillae.

Samples for RNA-seq and CUT&Tag were prepared as following: firstly, a quarter of the gonads in the XX36 group were processed for phenotypic sex identification. Three-quarters of the gonads in XX36 fish were processed for subsequent experiments. Fish from the XX28F, XY28M, XX36F, XX36P, and XY36M groups were collected at 10, 12, 15, and 20 dpf for subsequent experiments. After removing the head, tail, and viscera of the fry, the gonads were fixed in 4% paraformaldehyde (PFA) for IHC using the DMRT1 antibody (Abclonal, Cat# A8411 China), following Zhao et al. [54]. Additionally, pools of 30 pairs of embryonic gonads at 10 dpf and 12 dpf, and pools of 20 and 15 pairs of gonads at 15 dpf and 20 dpf, respectively, were preserved in TRIzol reagent for RNA-Seq. Fresh gonads were immediately processed using the CUT&Tag method following the experimental protocol. Three independent biological replicates were conducted for each of the temperature-phenotypic sex groups at four developmental stages sampled.

### RNA sequencing and gene differential expression analysis

Total RNA from three individuals of each temperature-sex groups was isolated using the TRIzol reagent (Life Technologies). RNA concentration and integrity were assessed for quality using the Qubit 2.0 Fluorometer (Invitrogen, USA) and the Fragment Analyzer 5400 (Agilent Technologies, CA, USA). Libraries were prepared with the NEBNext Ultra RNA Library Prep Kit and sequenced on an Illumina Novaseq 6000 platform with 150 bp paired-end reads, all performed by Novogene (Beijing China). After generating quality metrics with FastQC (v0.12.1) for each set of reads, the first 12 bases were trimmed with Trimmomatic (v0.39) [55] due to poor quality and abnormal base and k-mer distribution. Read pairs were then filtered again with Trimmomatic to remove all pairs in which at least one member had an average quality score below 20 or an N proportion above 5%. The reads from each replicate were mapped to the *O. niloticus* genome [56] and genes annotated using HISAT2 [57], and counted through edgeR [58] and normalized to the trimmed mean of M values (TMM) using Perl scripts provided by Trinity software [59]. PCA and hierarchical clustering analyses were also performed in R. The DESeq2 package from Bioconductor was used for differential gene expression analysis [60]. A threshold of *P* < 0.05 and fold change (FC) > 1 was required for candidacy as a differentially expressed gene (DEG). Gene expression heatmaps were visualized using the R package ComplexHeatmap [61]. The tilapia genes were annotated to the orthologous genes of zebrafish, and the corresponding zebrafish gene lists were used for the functional enrichment analyses. Enrichment analysis for the clusters was performed using the enricher function in the R package clusterProfiler (v4.7.1) [62]. Subsequently, REVIGO was used to reduce the redundancy of GO terms and summarize the results [63], using SimRel as a semantic similarity measure with a medium allowed similarity of 0.7. Additionally, we used DeepTools to calculate the average count for each base pair in I8. Next, the average count values for each base pair in exon 8 (E8) and exon 9 (E9) were computed. Finally, the ratio of the average count per base pair in I8 to the average counts per base pair in E8 and E9 was determined.

### CUT&Tag library preparation and sequencing

To obtain a cell suspension from a gonad tissue, a two-step enzymatic digestion was performed following Kossack et al. [64]. The gonads were mechanically dissected and incubated in a 2 ml low binding Eppendorf (EP) tube containing 1 mg/ml collagenase II and IV (Sigma-Aldrich, St. Louis, MO, USA) on a metal bath (28 °C for 25 min or 36 °C for 20 min, 200 r/min). The reaction was stopped by adding DMEM/F12 (Hyclone, Logan, UT, USA) with 10% fetal bovine serum (FBS) (Gibco; Thermo Fisher Scientific, Inc.), and the cells were pelleted at 600 g for 5 min. Single cells were obtained by gently pipetting, and resuspended in a 2 ml EP tube. Cells were counted under microscope by trypan blue staining.

CUT&Tag libraries were prepared using the CUT&Tag kit (Novoprotein, Suzhou, China). Briefly, approximately 3000–20,000 cells from each gonad were obtained and incubated with a 1:100 dilution of primary antibodies against IgG (Abcam, Cambridge, UK, ab313801), H3K4me2 (Abcam, ab32356), H3K4me3 (Abcam, ab213224), H3K9me2 (Abcam, ab176882), H3K9me3 (Abcam, ab8898), H3K27me2 (Abcam, ab24684), H3K27me3 (Cell Signaling, USA, C36B11), and H3K36me2 (Abcam, ab9049), and H3K36me3 (Abcam, ab282572) in a shaker overnight at 4 °C. Samples from the same group were combined and incubated with a 1:200 dilution of a Donkey Anti-Rabbit secondary antibody (Abcam, ab6701) at room temperature for 1 h. DNA was extracted using the beads adsorption method and indexed. The libraries were sequenced on the Illumina Novaseq 6000 system by Novogene (Beijing, China). We applied 150-bp paired-end sequencing with a sequencing depth of 3G base pairs of raw data. Two or three biological replicates were performed.

### CUT&Tag data analyses

Raw reads of CUT&Tag were first subjected to Trimmomatic (v0.39) [55] for adapter trimming. We performed a quality check using FastQC (v0.12.1) before alignment to ensure high-quality libraries. The paired-end sequencing reads were then aligned to the *O. niloticus* genome [56], followed by gene annotation using Bowtie 2 (v2.4.4) [65]. The BAM files from biological replicates were merged using Samtools (v1.14) [66] and converted into BigWig files using bamCoverage provided by DeepTools (v3.5.1) [67]. The BigWig files were visualized using DeepTools and IGV (v2.13.1) [68]. MACS2 (v2.2.7.1) [69] was used for peak calling. Peaks from all samples of the same mark were merged using GenomicRanges (v1.15.4) [70] in R to generate the reference peaks. Peaks were annotated to the Nile tilapia genome using the R package ChIPseeker (v1.36.0) [71]. The fragment counts for each peak were calculated using chromVAR (v1.8) [72]. The normalized count to trimmed mean of M values (CMM) was then calculated from fragment counts using Perl scripts provided by Trinity software [59]. The peak counts were used to identify differential peaks using the R package DESeq2 (v1.26.0) [60]. To assess correlations between transcriptome and epigenome data, Z-scaled TMM values averaged over all DEGs within each gene or cluster, and Z-scaled CMM values averaged over peaks annotated to promoter and genic regions of these DEGs were calculated. Pearson correlation coefficients (PCCs) were then calculated in R.

### CRISPR/Cas9–based knockout of *kdm6bb* and phenotypic sex identification

To investigate the phenotypic consequences of *kdm6bb* loss in tilapia, the CRISPR/Cas9 strategy was employed to generate a *kdm6bb* mutant tilapia line. Tilapia were maintained at 28 °C under a controlled light cycle (14 hours light, 10 hours dark) to induce spawning. Guide RNAs (gRNAs) were designed to target tilapia *kdm6bb* in exon 7 and exon 9 (S1 Data) according to Varshney et al. [73]. The generated gRNA template was used for *in vitro* transcription using the mMessage mMachine T7 Transcription Kit (Invitrogen) and purified using the RNA cleanup protocol from the RNAeasy Mini Kit (Qiagen, Hilden, Germany). Purified gRNA1 and gRNA2 (200 ng/µl each) were combined with Cas9 protein (800 ng/µl) (Genscript, Nanjing, China) in a 1:1:4 (by volume) ratio and subsequently injected into tilapia embryos (F0 fish) at the one-cell stage. These F0 fish were raised to maturity and genotyped using fin clipping. The two pairs of corresponding primers, *kdm6bb*-ko1 and *kdm6bb*-ko2 (S5A Fig and S1 Data), were used to screen founders with site mutations. The adult founders were outcrossed with wild-type fish to obtain F1 fish, which were subsequently genotyped and intercrossed to obtain F2 fish.

The wild-type and heterozygous F2 fish were treated with a high temperature of 36 °C at 6–20 dpf and then maintained at 28 °C until sampling. The gonads were collected at 10, 15, 20, 35, and 120 dpf following the procedures described above. Total RNA from the desired samples were extracted for qRT-PCR and the fins were used for genetic sex identification. The gonads were preserved in liquid nitrogen and 4% paraformaldehyde for subsequent experiments. Gonadal sections were stained with hematoxylin and eosin (H&E) (Biyuntian, China) following previously described methods [74]. Subsequently, the phenotypic sex of 60 tilapia at 120 dpf from each sex group (*kdm6bb*^+/+^ XX, *kdm6bb*^+/+^ XY, *kdm6bb*^+/−^ XX and *kdm6bb*^+/−^ XY for 28 °C and 36 °C, respectively) was identified by examining histological sections of the gonads stained with hematoxylin and eosin (H&E) under an optical microscope (Zeiss, Germany). The ratio of phenotypic sex was then calculated. This experiment was independently repeated three times.

### Tilapia brain neural (TBN) cells culture

TBN cells, obtained from the Institute of Hydrobiology, Chinese Academy of Sciences [75], were cultured in Leibovitz’s L-15 medium (Solaibao, Beijing, China) containing 20% FBS and 1% penicillin/streptomycin (both from Gibco) at 28 °C and 5% CO2 in a humidified atmosphere. Adherent cells were collected by incubation with 0.25% trypsin-EDTA. Cells were passaged and maintained at a density of 2.5 × 10^5 cells per ml.

### Quantitative RT-PCR

RNA was reverse-transcribed using the PrimeScript RT Reagent Kit (Takara, Kusatsu, Japan). The primers used for qRT-PCR are detailed in S1 Data. The specific steps for qRT-PCR mainly referred to the previous reference [25]. qRT-PCR assays were conducted in a 10 μL reaction volume using a Bio-Rad real-time PCR machine, following the SYBR Premix Ex Taq II protocol (Takara, Kusatsu, Japan). Relative RNA expression levels, normalized to *β-actin*, were determined using the comparative threshold cycle (Ct) method.

### Western blot

The polyclonal antibody against a fragment (S1 Text) of Kdm6bb was synthesized by Hua’an Biotech Co. Ltd. (Hangzhou, China). Total proteins from gonads were extracted, and their concentrations were determined using the BCA protein assay kit (Thermo). Equal amounts of Kdm6bb and H3K27me3 proteins were separated on 8% and 15% SDS-PAGE gels, respectively, and then transferred to PVDF membrane. The membranes were incubated overnight at 4°C with rabbit anti-Kdm6bb (custom made, 1:500), anti-H3K27me3 (Cell Signaling, 1:1000), and anti-β-actin antibodies (Hua’an, Cat# ET1702–67, 1:2000) respectively. They were then incubated at room temperature for 1.5 hours with a horseradish peroxidase (HRP)-labeled goat anti-rabbit secondary antibody (Hua’an, Cat# HA1001, 1:2000). Finally, the protein bands were visualized using an enhanced chemiluminescence reagent (Beyotime, Shanghai, China) in a Bio-Rad protein imaging system (CA, USA).

### Immunofluorescence (IF) and fluorescence *in situ* hybridization (FISH)

The gonads of fish were sampled at 10, 20, 35, and 120 dpf. TBN cells were divided into two groups and cultured at 28 °C and 36 °C, respectively. Immunofluorescence staining was performed to examine gene expression in tilapia gonads and cells. The gonadal sections were permeabilized with 0.3% Triton X-100 in PBS for 30 minutes and then blocked in 5% goat serum/PBS for 30 minutes at room temperature. The sections were then incubated with antibodies for Kdm6bb (custom made) and MYC (HuaAn, cat# R1208-1, 1:200) in 5% BSA/PBS overnight at 4°C. The HRP-labeled goat anti-rabbit secondary antibody (HuaAn, Cat# HA1001, 1:1000) was incubated for 1 h at room temperature to detect the primary antibodies. A tyramide signal amplification plus fluorescence system (TSA-Cy3) (Akoya Biosciences, Marlborough, MA, USA) was used according to the manufacturer’s instructions to detect the intensity. The nuclei were stained with DAPI.

Fluorescence *in situ* hybridization (FISH) was performed to examine gene expression in tilapia gonads. Digoxigenin and fluorescein-labeled RNA strands for *dmrt1* and *cyp19a1a* probes were transcribed in vitro from a linearized pGEMTeasy-target gene cDNA clone using the RNA labeling kit (Roche, Germany). The oligonucleotide primers for *in situ* hybridization are listed in S1 Data. For more sensitive fluorescence *in situ* hybridization detection, the tyramide signal amplification plus fluorescence system (TSA-FITC and TSA-Cy3) was processed followed the above steps. The nuclei were stained with DAPI. All IF and FISH sections were detected with confocal microscopy (Zeiss, Germany) at 514 nm (FITC) and 543 nm (Cy3).

### Plasmids construct, transfection, and transgenesis

The different isoforms of the *kdm6bb* gene in Nile tilapia were identified using RT-PCR in combination with the kd-sp primers (S1 Data). Total RNA was isolated from the gonads of 20 dpf fish, and reverse transcription was performed to prepare the cDNA. The two full-length *kdm6bb* transcripts: *kdm6bb*_I5_△I8 and *kdm6bb*_△I5_I8, including 5’ untranslated regions (5’UTR) and exons, were cloned by RT-PCR from the cDNA using *kdm6bb*_OE_F and *kdm6bb*_OE_R primers (S1 Data). Sanger sequencing was performed to identify the accuracy of the clones. Two plasmids, *pTol2-β-actin:kdm6bb_I5_△I8-myc, EGFP* (+ intron 5, - intron 8) and *pTol2-β-actin:kdm6bb_△I5_I8-myc, EGFP* (- intron 5, + intron 8), were obtained by integrating the full length *kdm6bb_I5_△I8* and *kdm6bb_△I5_I8* tagged with the myc coding sequence respectively into an linearized *pTol2-β-actin:P2A-EGFP* vector derived from Cao et al. [76] using homologous recombination (NEB, MA, USA) to make the expression of the myc-tagged Kdm6bb variants under the control of fish *β-actin* promoter. Additionally, the *pTol2-β-actin:kdm6bb_△I5_△I8-myc, EGFP* （- intron 5, - intron 8） plasmid was acquired by deleting intron 8 from the *pTol2-β-actin:kdm6bb_△I5_I8-myc, EGFP* plasmid. The nucleotide sequences of the three *kdm6bb* variants were aligned and shown in S1 Text.

Three μg of each plasmid was transfected into 2 × 10^5 TBN cells using the Amaxa 4D-Nucleofector System (Lonza, Walkersville MD, USA) under electroporation program CM-137. The cells were then divided into two groups and cultured at 28 °C and 36 °C, respectively. Four days post-transfection, immunoblotting and immunofluorescence staining were performed on the cells using an anti-MYC antibody (HuaAn) to detect the expression of exogenous Kdm6bb.

To study the function of the *kdm6bb* variants in Tilapia sex determination, the plasmids were dissolved in water to a final concentration of 50 ng/ µ L. Each DNA construct was mixed with an equal volume of Tol2 transposase mRNA (50 ng/ µ L) transcribed *in vitro* from the pCMV-Tol2 transposase plasmid using the mMESSAGE mMACHINE SP6 Transcription Kit (Thermo). Approximately 1 nL of the DNA/mRNA mixture was injected into Nile tilapia eggs at the one-cell stage. The injected eggs were cultivated at 28 °C, and the fish with the transgene was identified by examining EGFP expression using a fluorescence microscope (Zeiss). The phenotypic sex was determined by analysis of *dmrt1* and *cyp19a1a* expression in gonads at 20 dpf. Additionally, the gonads were subjected to immunofluorescence staining using an anti-MYC primary antibody. The relative expression levels of male-related genes including *dmrt1*, *gsdf*, *amh*, and *sox8a* as well as female-related genes including *foxl2*, *foxl3*, *cyp19a1a*, *fsta*, and *wt1a*, were quantified by qRT-PCR in the gonads of the three transgene groups using primers listed in S1 Data.

### Statistics

All data are expressed as the mean ± SEM and were analyzed using one-way analysis of variance (ANOVA) with Tukey’s post-test in SPSS version 21.0 software (IBM, Chicago, USA). Asterisks indicate statistically significant differences between groups (*P < 0.05, **P < 0.01, ***P < 0.001). The number of biological samples (n) is stated in the Figure legends. Three biological samples were used for RNA-Seq, CUT&Tag, qRT-PCR, immunoblotting, H&E staining, IF, IHC, and FISH. For male ratio calculation, biological samples (n) represent the individual numbers for each family, and three families from different parents were developed for each group (*kdm6bb*^+/+^XX, *kdm6bb*^+/-^XX, *kdm6bb*^+/+^XY, *kdm6bb*^+/-^XY for 28 °C and 36 °C, *kdm6bb*_I5_△I8, *kdm6bb*_△I5_I8, and *kdm6bb_*△I5_△I8). The expression of Kdm6bb protein in the nucleus and cytoplasm of gonads was quantified using ImageJ software (LOCI, University of Wisconsin, USA) to determine the proportion relative to the total nuclear area. The numerical data represent the average of six measurements.

## Supporting information

S1 Fig PCA of the RNA-seq samples (A), heatmap showing altered gene expression in male-biased and female-biased clusters (B) and key players in sex determination and TSR: Key players adapted from previous studies, showing significant expression changes (C) [32–35,50]. (TIF)

S2 Fig An epigenetic atlas of high temperature-induced masculinization in Nile tilapia. (A) Average number of peaks detected for each of the eight epigenetic marks across different genotype-phenotype sex combinations. (B) Percentage of genome coverage detected for each of the eight epigenetic marks across different genotype-phenotype sex combinations. (C) PCA analysis for each of the eight epigenetic marks across different genotype-phenotype sex combinations. (D) Genome-wide distribution of peaks for epigenetic marks. (E) Profile of epigenetic marks along genic regions for each genotype-phenotype sex combination. TSS: Transcription Start Site; TES: Transcription End Site.
(TIF)

S3 Fig Chromatin dynamics coordinate transcription profiles during high temperature-induced masculinization. (A-B) Heatmap displaying transcriptional dynamics and the level of modifications of H3K27me2 (A) and H3K4me2 (B) for the temperature responsive genes, with PCC below -0.5 for H3K27me3 and above 0.5 for H3K4me3. The left side lists the representative genes known to be involved in sex determination. (C-D) Dynamic transcription (top), H3K27me2/3 (C), and H3K4me2/3 (D) modification tracks (bottom) for sex differentiating genes. Gene expression data are shown as mean ± SEM of three biological replicates. The data points derived from the same individual are denoted by the same color.
(TIF)

S4 Fig The expression of epigenetic factor genes during high temperature-induced masculinization. (A) Heatmap showing the expression levels of epigenetic factor genes regulated by high temperature. (B-E) Expression patterns of representative genes in different genotype-phenotype sex groups at four developmental stages. The data points derived from the same individual are denoted by the same color.
(TIF)

S5 Fig Loss of *kdm6bb* leads to male-to-female sex reversal in XY Nile tilapia raised at 28 °C. (A)Schematic of gRNA and primers design used for *kdm6bb* knockout and Identification of *kdm6bb* knockout tilapia via PCR and electrophoresis. (B)DNA sequencing of the *kdm6bb* mutant allele, illustrating the deleted sequence highlighted in a blue box and a 15-bp insertion indicated in a red box, both introduced by CRISPR/Cas9. (C)The cumulative proportion surviving (%) of various genotypes of offspring from *kdm6bb*^+/-^ parents. Both heterozygous and homozygous *kdm6bb* mutants in Nile tilapia exhibited partial or nearly complete mortality. (D)qRT-PCR analysis of *gsdf*, *amh*, and *foxl2* in gonads at five developmental stages. The data points derived from the same individual are denoted by the same color.
(TIF)

S6 Fig Identification of the *kdm6bb* transcript variant conferring high temperature-induced sex reversal. (A) Screenshots of *kdm6bb* RNA-Seq data in tilapia brains displayed using IGV browser. (B) Gel electrophoresis of PCR on *kdm6bb* cDNA. Gel electrophoresis (130 V, 2 h) was carried out in 3% agarose. (C) Relative percentage of the *kdm6bb*_I5_△I8 isoform compared to the total count of *kdm6bb* transcripts in gonads at 10, 12, 15, and 20 dpf stages for XX36F and XX36P. The data points derived from the same individual are denoted by the same color. (D) RT-PCR gel electrophoresis with *dmrt1* and *cyp19a1a* genes to identify the phenotypic sex of transgenic gonads at 20 dpf overexpressing one of the three *kdm6bb* isoforms at 28 °C. (E) Sex reversal ratio (percentage of testis) of gonads overexpressing one of the three *kdm6bb* isoforms at 28 °C. Gonadal sex was determined by RT-PCR analysis of *dmrt1* and *cyp19a1a* expression levels in gonads at 20 dpf. EGFP, enhanced green fluorescent protein. (F) Relative expression of *fsta*, *wt1a*, *fancl*, and *sox8a* in transgenic fish expressing one of the three *kdm6bb* constructs raised at 28 °C. The data points derived from the same individual are denoted by the same color.
(TIF)

S1 Text Nuclear sequence alignment of *kdm6bb*_△I5_I8, *kdm6bb*_△I5_△I8, and *kdm6bb*_I5_△I8 cDNA with translation initiation codon bolded and underlined. (DOC)

S1 Data List of nucleotide/amino acid sequence used in this study. (XLSX)

S2 Data The average alignment and filtering retention rate of the samples. (XLSX)

S3 Data Genes and gene expression levels in Temp-up and Temp-down clusters. (XLSX)

S4 Data Genes levels in female-biased and male-biased clusters. (XLSX)

S5 Data KEGG and GO enrichment analyses were conducted on the genes in the Temp-up and Temp-down cluster. (XLSX)

S6 Data Genes and gene expression levels in M-T_up_, M-T_down_, F-T_up_, and F-T_down_ clusters. (XLSX)

S7 Data The genes within the Temp-up and Temp-down clusters contain peaks associated with various marks. (XLSX)

S8 Data The expression of genes regulated (|cor| > 0.5) by corresponding marks. (XLSX)

S9 Data KEGG and GO enrichment analyses were conducted on the genes regulated by the corresponding marks. (XLSX)
