# Supplementary figures and images for: Heat inducible nuclear translocation of Kdm6bb drives temperature dependent sex reversal in Nile tilapia
Source: PLoS Genet. 2025 Apr 30;21(4):e1011664. doi: 10.1371/journal.pgen.1011664 (PMC12043187; doi:10.1371/journal.pgen.1011664)

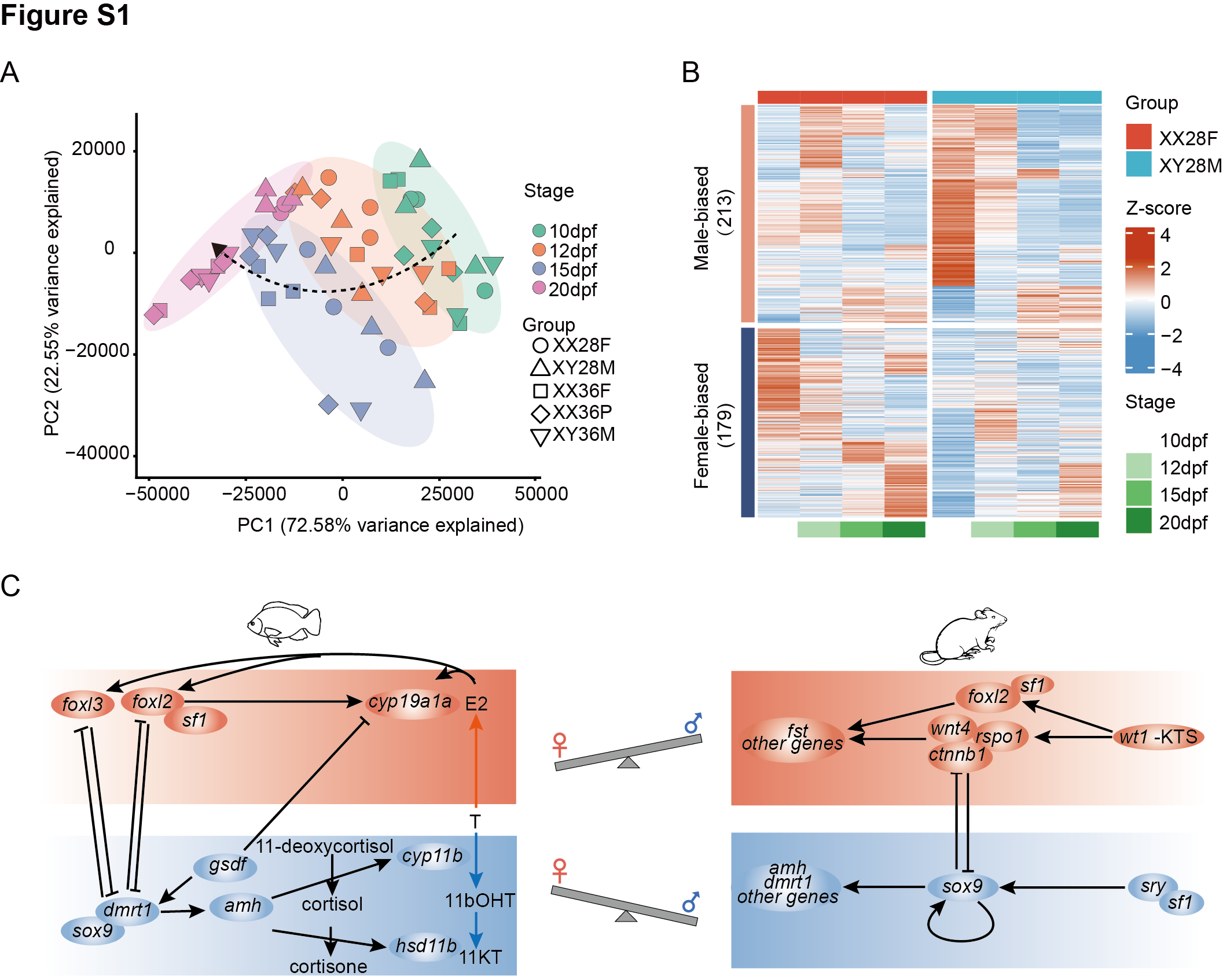

Supplement: S1 Fig — (TIF) [file pgen.1011664.s001.tif]

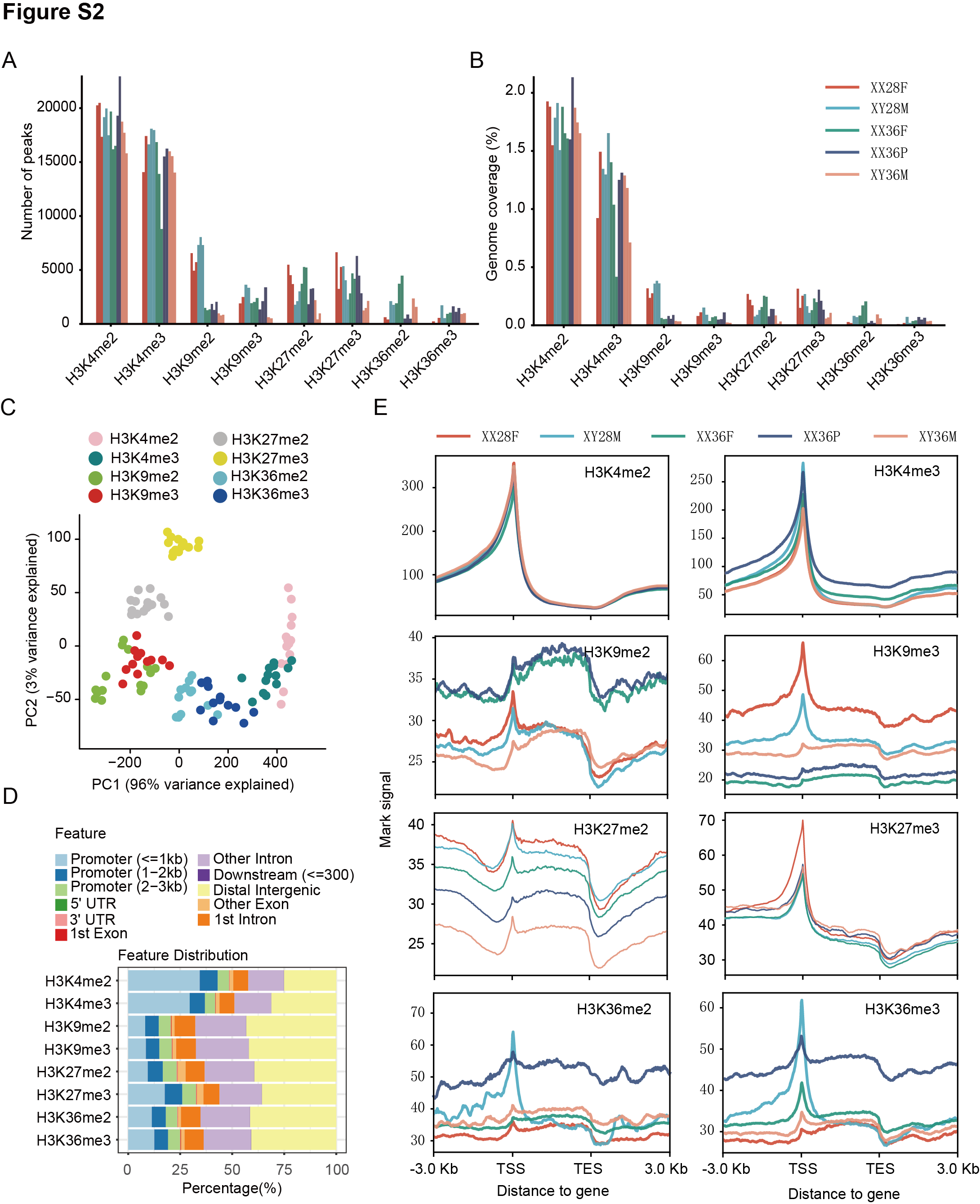

Supplement: S2 Fig — (A) Average number of peaks detected for each of the eight epigenetic marks across different genotype-phenotype sex combinations. (B) Percentage of genome coverage detected for each of the eight epigenetic marks across different genotype-phenotype sex combinations. (C) PCA analysis for each of the eight epigenetic marks across different genotype-phenotype sex combinations. (D) Genome-wide distribution of peaks for epigenetic marks. (E) Profile of epigenetic marks along genic regions for each genotype-phenotype sex combination. TSS: Transcription Start Site; TES: Transcription End Site. (TIF) [file pgen.1011664.s002.tif]

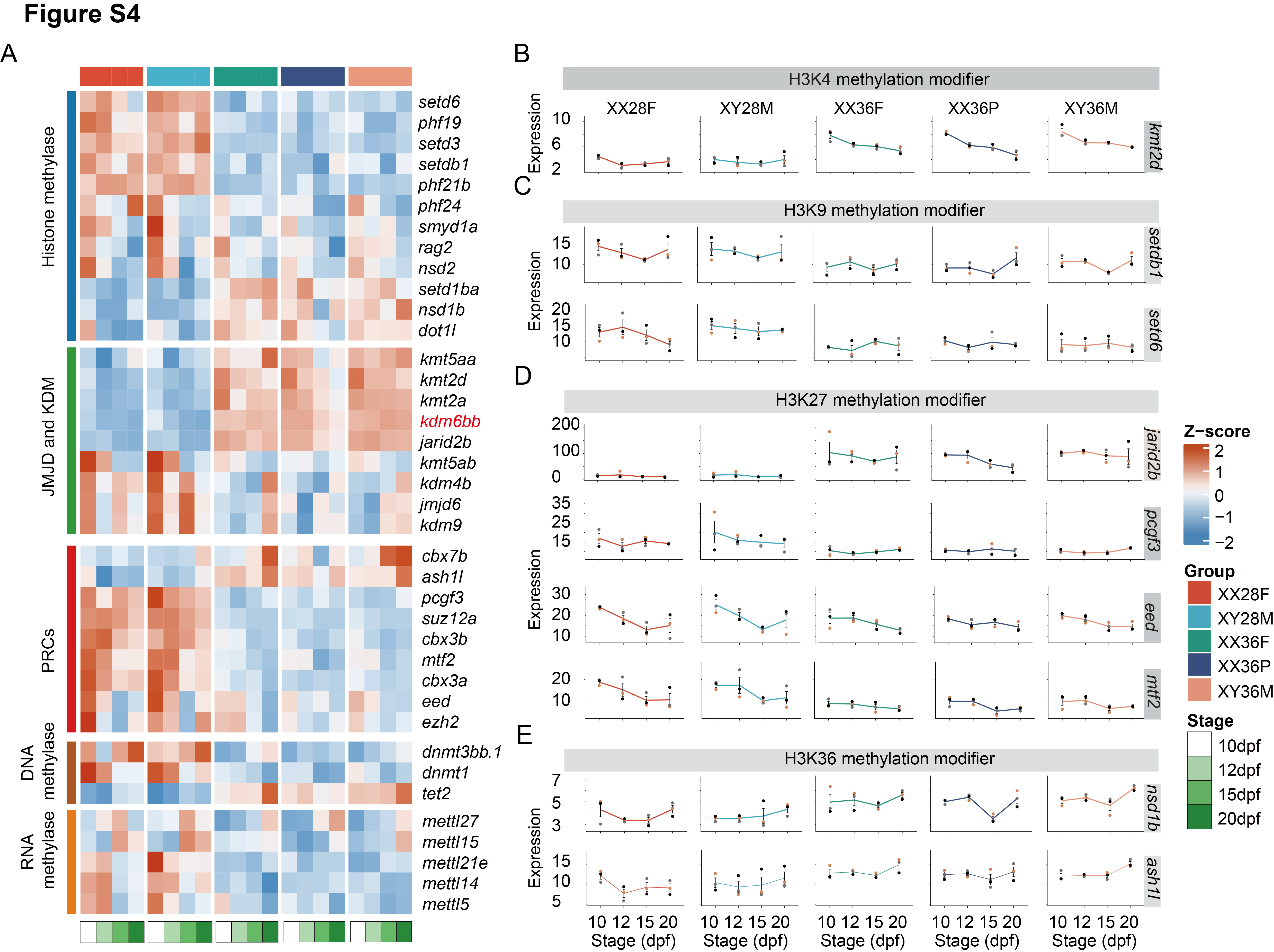

Supplement: S3 Fig — (A-B) Heatmap displaying transcriptional dynamics and the level of modifications of H3K27me2 (A) and H3K4me2 (B) for the temperature responsive genes, with PCC below -0.5 for H3K27me3 and above 0.5 for H3K4me3. The left side lists the representative genes known to be involved in sex determination. (C-D) Dynamic transcription (top), H3K27me2/3 (C), and H3K4me2/3 (D) modification tracks (bottom) for sex differentiating genes. Gene expression data are shown as mean ± SEM of three biological replicates. The data points derived from the same individual are denoted by the same color. (TIF) [file pgen.1011664.s003.tif]

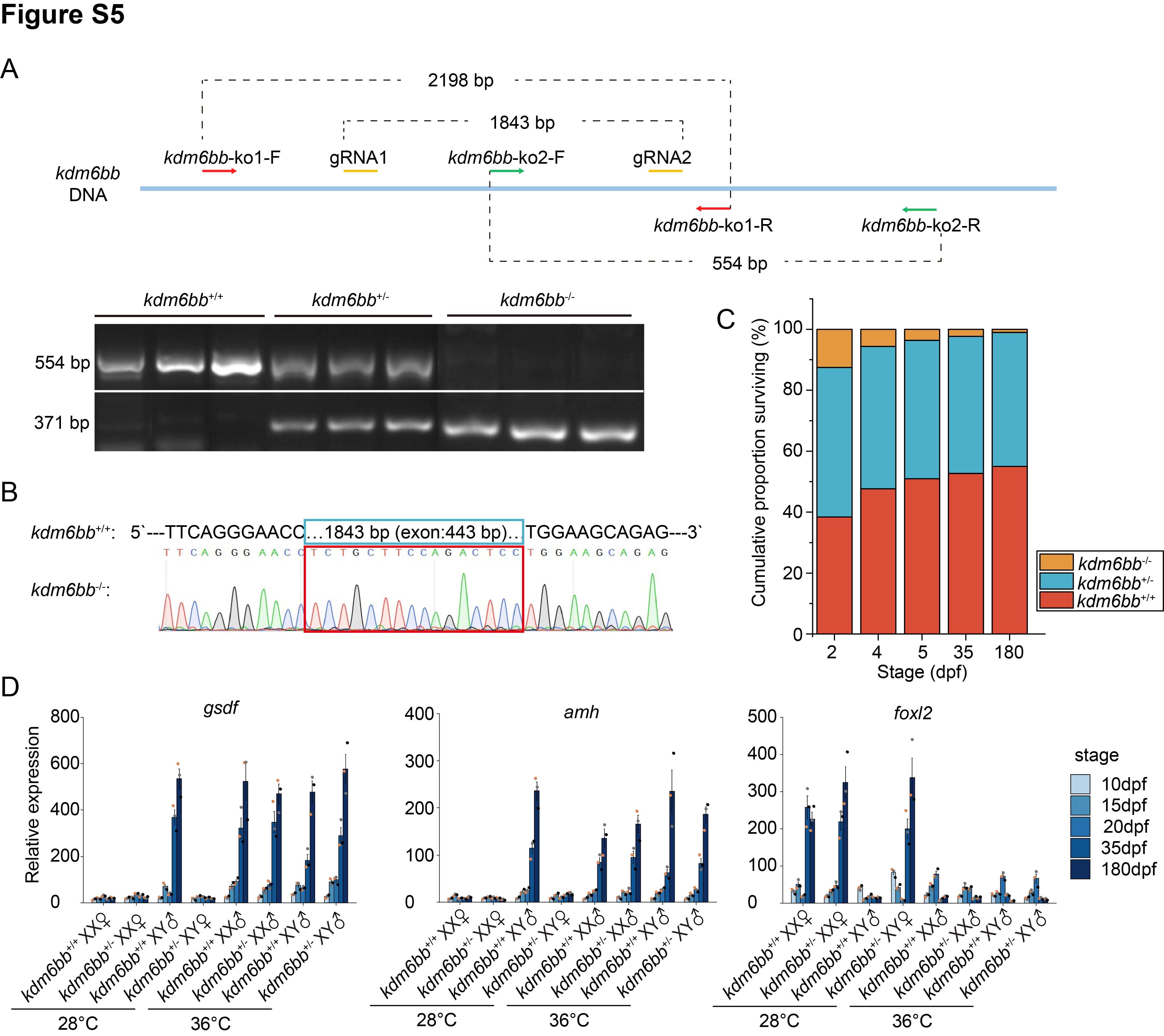

Supplement: S5 Fig — (A)Schematic of gRNA and primers design used for kdm6bb knockout and Identification of kdm6bb knockout tilapia via PCR and electrophoresis. (B)DNA sequencing of the kdm6bb mutant allele, illustrating the deleted sequence highlighted in a blue box and a 15-bp insertion indicated in a red box, both introduced by CRISPR/Cas9. (C)The cumulative proportion surviving (%) of various genotypes of offspring from kdm6bb+/- parents. Both heterozygous and homozygous kdm6bb mutants in Nile tilapia exhibited partial or nearly complete mortality. (D)qRT-PCR analysis of gsdf, amh, and foxl2 in gonads at five developmental stages. The data points derived from the same individual are denoted by the same color. (TIF) [file pgen.1011664.s005.tif]

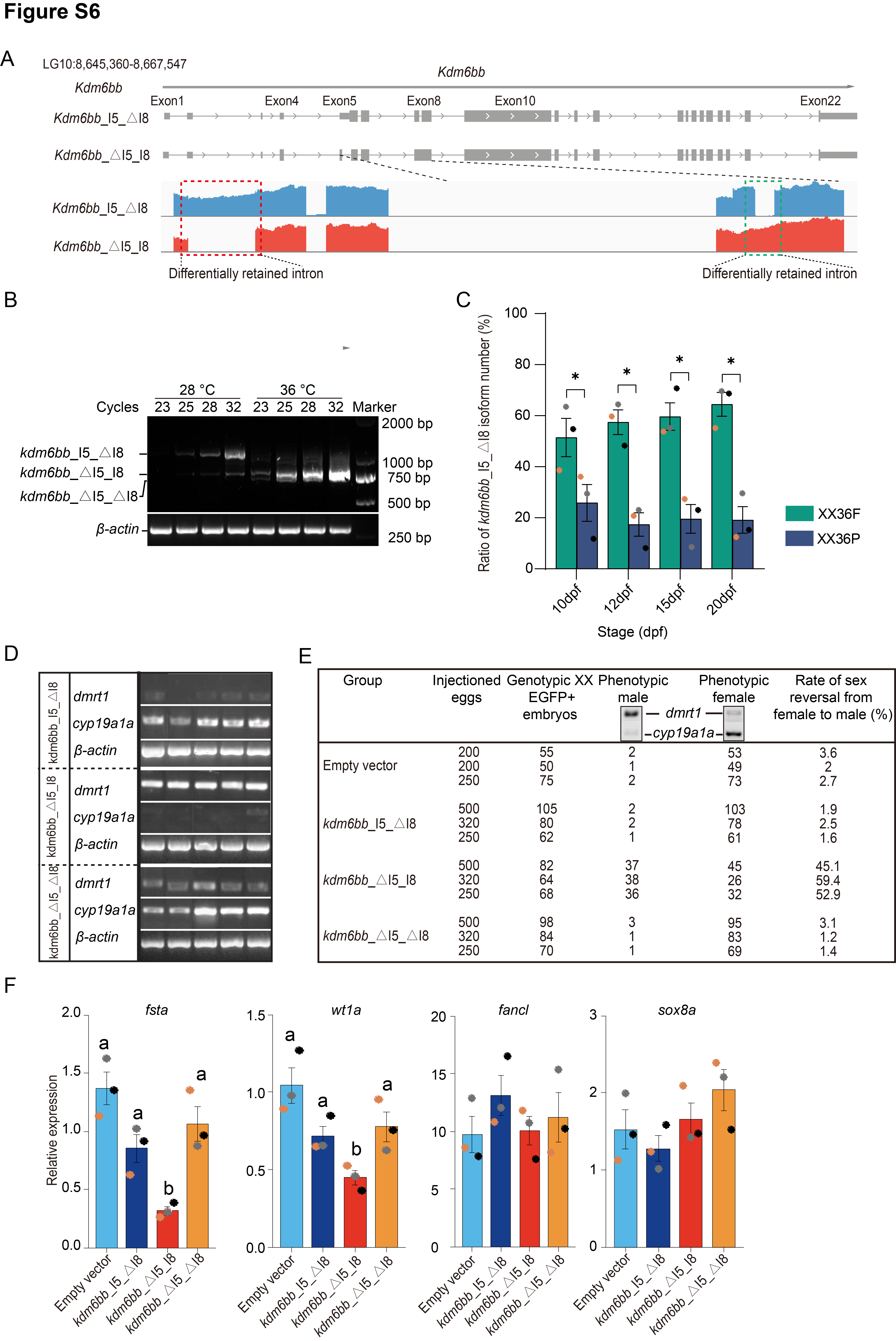

Supplement: S6 Fig — (A) Screenshots of kdm6bb RNA-Seq data in tilapia brains displayed using IGV browser. (B) Gel electrophoresis of PCR on kdm6bb cDNA. Gel electrophoresis (130 V, 2 h) was carried out in 3% agarose. (C) Relative percentage of the kdm6bb_I5_△I8 isoform compared to the total count of kdm6bb transcripts in gonads at 10, 12, 15, and 20 dpf stages for XX36F and XX36P. The data points derived from the same individual are denoted by the same color. (D) RT-PCR gel electrophoresis with dmrt1 and cyp19a1a genes to identify the phenotypic sex of transgenic gonads at 20 dpf overexpressing one of the three kdm6bb isoforms at 28 °C. (E) Sex reversal ratio (percentage of testis) of gonads overexpressing one of the three kdm6bb isoforms at 28 °C. Gonadal sex was determined by RT-PCR analysis of dmrt1 and cyp19a1a expression levels in gonads at 20 dpf. EGFP, enhanced green fluorescent protein. (F) Relative expression of fsta, wt1a, fancl, and sox8a in transgenic fish expressing one of the three kdm6bb constructs raised at 28 °C. The data points derived from the same individual are denoted by the same color. (TIF) [file pgen.1011664.s006.tif]
