# Supplementary material for: Heat inducible nuclear translocation of Kdm6bb drives temperature dependent sex reversal in Nile tilapia
Source: PLoS Genet. 2025 Apr 30;21(4):e1011664. doi: 10.1371/journal.pgen.1011664 (PMC12043187; doi:10.1371/journal.pgen.1011664)
Supplement: S1 Text — (DOC) [file pgen.1011664.s007.doc]

**S1 Text. Nuclear sequence alignment of *kdm6bb*_△I5_I8, *kdm6bb*_△I5_△I8, and *kdm6bb*_ I5_△I8 cDNA with translation initiation codon bolded and underlined.**

| Group Sequence |
| --- |
| *kdm6bb*_△I5_ I8 AAAAAGGGGAACAGAGGGGCGTGCGGACGAGAGAGAACCAAAACAAAAACAACAAAAAAT  *kdm6bb*_△I5_△I8 AAAAAGGGGAACAGAGGGGCGTGCGGACGAGAGAGAACCAAAACAAAAACAACAAAAAAT  *kdm6bb*_ I5_ △I8 AAAAAGGGGAACAGAGGGGCGTGCGGACGAGAGAGAACCAAAACAAAAACAACAAAAAAT  ************************************************************  *kdm6bb*_△I5_ I8 AGAAGCTAGCCAAGGATGAGGATGTCTATTTAGAGGGATCCAAGACTCCGCGGGTCTGCT  *kdm6bb*_△I5_△I8 AGAAGCTAGCCAAGGATGAGGATGTCTATTTAGAGGGATCCAAGACTCCGCGGGTCTGCT  *kdm6bb*_ I5_ △I8 AGAAGCTAGCCAAGGATGAGGATGTCTATTTAGAGGGATCCAAGACTCCGCGGGTCTGCT  ************************************************************  *kdm6bb*_△I5_ I8 GTCTTTCTATTCCTGCCTGACAGCATCATCTAATTTGGACCAGAAAGTCCCCACTCCCAC  *kdm6bb*_△I5_△I8 GTCTTTCTATTCCTGCCTGACAGCATCATCTAATTTGGACCAGAAAGTCCCCACTCCCAC  *kdm6bb*_ I5_ △I8 GTCTTTCTATTCCTGCCTGACAGCATCATCTAATTTGGACCAGAAAGTCCCCACTCCCAC  ************************************************************  *kdm6bb*_△I5_ I8 TCACCCCCAACTCCACCAACAACGCAGTTCTCTCTGCATTTATTTTGAAGACTGCTTTTT  *kdm6bb*_△I5_△I8 TCACCCCCAACTCCACCAACAACGCAGTTCTCTCTGCATTTATTTTGAAGACTGCTTTTT  *kdm6bb*_ I5_△I8 TCACCCCCAACTCCACCAACAACGCAGTTCTCTCTGCATTTATTTTGAAGACTGCTTTTT  ************************************************************  *kdm6bb*_△I5_ I8 TTGCTCTCAGGTGTGAAGTGGAGGGCCTCCAAAACACAGACCAAGACTGGCTGC**ATG**TAT  *kdm6bb*_△I5_△I8 TTGCTCTCAGGTGTGAAGTGGAGGGCCTCCAAAACACAGACCAAGACTGGCTGC**ATG**TAT  *kdm6bb*_ I5_△I8 TTGCTCTCAGGTGTGAAGTGGAGGGCCTCCAAAACACAGACCAAGACTGGCTGCATGTAT  ************************************************************  *kdm6bb*_△I5_ I8 CACTCAGTAGAGCTCTACTCTGGCCGTAACACATGGGACTCCTACTCAGTTGGAGGACCT  *kdm6bb*_△I5_△I8 CACTCAGTAGAGCTCTACTCTGGCCGTAACACATGGGACTCCTACTCAGTTGGAGGACCT  *kdm6bb*_ I5_△I8 CACTCAGTAGAGCTCTACTCTGGCCGTAACACATGGGACTCCTACTCAGTTGGAGGACCT  ************************************************************  *kdm6bb*_△I5_ I8 AACAAAGGACAATGGGCTCCTGTAAATGCTCGCCCCTGGAGCCACACACAGAGGCTGCAA  *kdm6bb*_△I5_△I8 AACAAAGGACAATGGGCTCCTGTAAATGCTCGCCCCTGGAGCCACACACAGAGGCTGCAA  *kdm6bb*_ I5_△I8 AACAAAGGACAATGGGCTCCTGTAAATGCTCGCCCCTGGAGCCACACACAGAGGCTGCAA  ************************************************************  *kdm6bb*_△I5_ I8 GAAGGGCGCAAACAGCCATATCATTCTCTGTCAAGTCGTATTTATAGCAG----------  *kdm6bb*_△I5_△I8 GAAGGGCGCAAACAGCCATATCATTCTCTGTCAAGTCGTATTTATAGCAG----------  *kdm6bb*_ I5_△I8 GAAGGGCGCAAACAGCCATATCATTCTCTGTCAAGTCGTATTTATAGCAGGTAAGTATTT  **************************************************  *kdm6bb*_△I5_ I8 ------------------------------------------------------------  *kdm6bb*_△I5_△I8 ------------------------------------------------------------  *kdm6bb*_ I5_△I8 ATCATTTATACTACACTTAAAAAGCTTTCGTGTTGACCAGCTTTCAGAGTTATGTGCTGT    *kdm6bb*_△I5_ I8 ------------------------------------------------------------  *kdm6bb*_△I5_△I8 ------------------------------------------------------------  *kdm6bb*_ I5_△I8 GATTTTTTTGTTACTGAAACTGCTTTTGTCAGTGTCATCTTCTCTAAGCTGGCTTCACTC    *kdm6bb*_△I5_ I8 ------------------------------------------------------------  *kdm6bb*_△I5_△I8 ------------------------------------------------------------  *kdm6bb*_ I5_△I8 GTATTGAGAAACCGAACAGTACAACTCTTGATGCCCTCGTGGATATGAGTATTGACTGGA    *kdm6bb*_△I5_ I8 ------------------------------------------------------------  *kdm6bb*_△I5_△I8 ------------------------------------------------------------  *kdm6bb*_ I5_△I8 AAAGTTTCTGTTGTACCGTGGTGTAATTACC**ATG**AATGCCTGTCCACAGTGTGGTCCTGA    *kdm6bb*_△I5_ I8 ---------------------GGGTGACAGAACAATCAACCATGTCCCGGACAAAGGTAT  *kdm6bb*_△I5_△I8 ---------------------GGGTGACAGAACAATCAACCATGTCCCGGACAAAGGTAT  *kdm6bb*_ I5_△I8 GTTTAGTTGTTTTCACAACAGGGGTGACAGAACAATCAACCATGTCCCGGACAAAGGTAT  ***************************************  *kdm6bb*_△I5_ I8 CTCAAAGGGGCATAGCCAGCTGCTGCGAATTTGGGATGGTAAAGAGCTGCAGTTTGAGGT  *kdm6bb*_△I5_△I8 CTCAAAGGGGCATAGCCAGCTGCTGCGAATTTGGGATGGTAAAGAGCTGCAGTTTGAGGT  *kdm6bb*_ I5_△I8 CTCAAAGGGGCATAGCCAGCTGCTGCGAATTTGGGATGGTAAAGAGCTGCAGTTTGAGGT  ************************************************************  *kdm6bb*_△I5_ I8 CCAGAACTGGCATCACAACTCCATCCGTTCGTTCCACATCCGAGATGGCACCAACAATGG  *kdm6bb*_△I5_△I8 CCAGAACTGGCATCACAACTCCATCCGTTCGTTCCACATCCGAGATGGCACCAACAATGG  *kdm6bb*_ I5_△I8 CCAGAACTGGCATCACAACTCCATCCGTTCGTTCCACATCCGAGATGGCACCAACAATGG  ************************************************************  *kdm6bb*_△I5_ I8 TTATCTGCCAGGACCAGAGGAACGCTACGCAAACTGGCAAAACAATAACGGGGGCCCTCG  *kdm6bb*_△I5_△I8 TTATCTGCCAGGACCAGAGGAACGCTACGCAAACTGGCAAAACAATAACGGGGGCCCTCG  *kdm6bb*_ I5_△I8 TTATCTGCCAGGACCAGAGGAACGCTACGCAAACTGGCAAAACAATAACGGGGGCCCTCG  ************************************************************  *kdm6bb*_△I5_ I8 CCTGCATCGCAACAACAGGGAGCTTCAGGGAACCGCGCCAGAGAGATGGGCCCACTCAGA  *kdm6bb*_△I5_△I8 CCTGCATCGCAACAACAGGGAGCTTCAGGGAACCGCGCCAGAGAGATGGGCCCACTCAGA  *kdm6bb*_ I5_△I8 CCTGCATCGCAACAACAGGGAGCTTCAGGGAACCGCGCCAGAGAGATGGGCCCACTCAGA  ************************************************************  *kdm6bb*_△I5_ I8 CCCCCGCAGGAGTTTCCCAGACAGGATGGTGAACAACAGAACAGGACCATGGAAACGGCC  *kdm6bb*_△I5_△I8 CCCCCGCAGGAGTTTCCCAGACAGGATGGTGAACAACAGAACAGGACCATGGAAACGGCC  *kdm6bb*_ I5_△I8 CCCCCGCAGGAGTTTCCCAGACAGGATGGTGAACAACAGAACAGGACCATGGAAACGGCC  ************************************************************  *kdm6bb*_△I5_ I8 AGCCCTCCACCAGCGACGGGAGCAAGCGCATCACCACAGTCCACCCCCGGAGCACCCATT  *kdm6bb*_△I5_△I8 AGCCCTCCACCAGCGACGGGAGCAAGCGCATCACCACAGTCCACCCCCGGAGCACCCATT  *kdm6bb*_ I5_△I8 AGCCCTCCACCAGCGACGGGAGCAAGCGCATCACCACAGTCCACCCCCGGAGCACCCATT  ************************************************************  *kdm6bb*_△I5_ I8 AACCCCCCGGGATGAGTGTCCAGCTAAGAGGAGAAGAGACTCGGGACCTGATCAGGCTTG  *kdm6bb*_△I5_△I8 AACCCCCCGGGATGAGTGTCCAGCTAAGAGGAGAAGAGACTCGGGACCTGATCAGGCTTG  *kdm6bb*_ I5_△I8 AACCCCCCGGGATGAGTGTCCAGCTAAGAGGAGAAGAGACTCGGGACCTGATCAGGCTTG  ************************************************************  *kdm6bb*_△I5_ I8 TCATACTGGATCCAGGCACTTACCTTTACTTGCCCATGCCCCATCATCACCTCACCAACA  *kdm6bb*_△I5_△I8 TCATACTGGATCCAGGCACTTACCTTTACTTGCCCATGCCCCATCATCACCTCACCAACA  *kdm6bb*_ I5_△I8 TCATACTGGATCCAGGCACTTACCTTTACTTGCCCATGCCCCATCATCACCTCACCAACA  ************************************************************  *kdm6bb*_△I5_ I8 GCGCTCCAACCAGGATGATTGGAAGCCTCCAAGTGACAGGGCGGGTCCATGCCACCACTC  *kdm6bb*_△I5_△I8 GCGCTCCAACCAGGATGATTGGAAGCCTCCAAGTGACAGGGCGGGTCCATGCCACCACTC  *kdm6bb*_ I5_△I8 GCGCTCCAACCAGGATGATTGGAAGCCTCCAAGTGACAGGGCGGGTCCATGCCACCACTC  ************************************************************  *kdm6bb*_△I5_ I8 TGAACACAGGACCTCAACAACACAGCAACAGGTGAGCATGTACAATTTGTATTATTCCTC  *kdm6bb*_△I5_△I8 TGAACACAGGACCTCAACAACACAGCAACAG-----------------------------  *kdm6bb*_ I5_△I8 TGAACACAGGACCTCAACAACACAGCAACAG-----------------------------  *******************************  *kdm6bb*_△I5_ I8 AGATTTGGCACAACCATTGATTTCCAAATCTGGCTTTTCACTTTTGCAGGAAACCTCTAA  *kdm6bb*_△I5_△I8 -------------------------------------------------GAAACCTCTAA  *kdm6bb*_ I5_△I8 -------------------------------------------------GAAACCTCTAA  ***********  *kdm6bb*_△I5_ I8 ACTGCGAGCTGGGAGACACGGCTTCAGTGACCCAGTGCCGTCCAACCAGAGCTGCGGCAG  *kdm6bb*_△I5_△I8 ACTGCGAGCTGGGAGACACGGCTTCAGTGACCCAGTGCCGTCCAACCAGAGCTGCGGCAG  *kdm6bb*_ I5_△I8 ACTGCGAGCTGGGAGACACGGCTTCAGTGACCCAGTGCCGTCCAACCAGAGCTGCGGCAG  ************************************************************  *kdm6bb*_△I5_ I8 GAGGCCACCACATTATGGAAGCAGAGGGAAGGTCGAACGGAAAATCTCATCTTCACCAGC  *kdm6bb*_△I5_△I8 GAGGCCACCACATTATGGAAGCAGAGGGAAGGTCGAACGGAAAATCTCATCTTCACCAGC  *kdm6bb*_ I5_△I8 GAGGCCACCACATTATGGAAGCAGAGGGAAGGTCGAACGGAAAATCTCATCTTCACCAGC  ************************************************************  *kdm6bb*_△I5_ I8 AGACCACACCCGTGTGCCTTACAGCCACCAACACCATCACTATAACCATCAACGGCACAC  *kdm6bb*_△I5_△I8 AGACCACACCCGTGTGCCTTACAGCCACCAACACCATCACTATAACCATCAACGGCACAC  *kdm6bb*_ I5_△I8 AGACCACACCCGTGTGCCTTACAGCCACCAACACCATCACTATAACCATCAACGGCACAC  ************************************************************  *kdm6bb*_△I5_ I8 AGGCCAGCCTAGAGCACCTCAGCACAACGCCCCACTACACCCTCTGGAGGATAAGGACTC  *kdm6bb*_△I5_△I8 AGGCCAGCCTAGAGCACCTCAGCACAACGCCCCACTACACCCTCTGGAGGATAAGGACTC  *kdm6bb*_ I5_△I8 AGGCCAGCCTAGAGCACCTCAGCACAACGCCCCACTACACCCTCTGGAGGATAAGGACTC  ************************************************************  *kdm6bb*_△I5_ I8 TTGGAACCATCATCACAAACAAAGCACAGAGAATCAGCGCATTCATCAACGAACAAATTC  *kdm6bb*_△I5_△I8 TTGGAACCATCATCACAAACAAAGCACAGAGAATCAGCGCATTCATCAACGAACAAATTC  *kdm6bb*_ I5_△I8 TTGGAACCATCATCACAAACAAAGCACAGAGAATCAGCGCATTCATCAACGAACAAATTC  ************************************************************  *kdm6bb*_△I5_ I8 AAGGGACCCCTCCCTCTCCAAATCTGGTGTAGACTCTCCTCAGTACTCATCTGTCCTCTG  *kdm6bb*_△I5_△I8 AAGGGACCCCTCCCTCTCCAAATCTGGTGTAGACTCTCCTCAGTACTCATCTGTCCTCTG  *kdm6bb*_ I5_△I8 AAGGGACCCCTCCCTCTCCAAATCTGGTGTAGACTCTCCTCAGTACTCATCTGTCCTCTG  ************************************************************  *kdm6bb*_△I5_ I8 TGGTCCGAACAATGGAGGTTCTACTTCCAGCAGTACAGGTGCTCCTTCCAGTCCTTCTTC  *kdm6bb*_△I5_△I8 TGGTCCGAACAATGGAGGTTCTACTTCCAGCAGTACAGGTGCTCCTTCCAGTCCTTCTTC  *kdm6bb*_ I5_△I8 TGGTCCGAACAATGGAGGTTCTACTTCCAGCAGTACAGGTGCTCCTTCCAGTCCTTCTTC  ************************************************************  *kdm6bb*_△I5_ I8 AGACAAAATGCTCAATGGCAGAAAAGATTCACCAGTCATTCAGCAGCGTTGTCCCAGCCT  *kdm6bb*_△I5_△I8 AGACAAAATGCTCAATGGCAGAAAAGATTCACCAGTCATTCAGCAGCGTTGTCCCAGCCT  *kdm6bb*_ I5_△I8 AGACAAAATGCTCAATGGCAGAAAAGATTCACCAGTCATTCAGCAGCGTTGTCCCAGCCT  ************************************************************  *kdm6bb*_△I5_ I8 CAGTGGGCAGCTAAAGCTCCAAGAAAGTCCCAGTGTCATTCCGAGACCCAGTCGGGCATC  *kdm6bb*_△I5_△I8 CAGTGGGCAGCTAAAGCTCCAAGAAAGTCCCAGTGTCATTCCGAGACCCAGTCGGGCATC  *kdm6bb*_ I5_△I8 CAGTGGGCAGCTAAAGCTCCAAGAAAGTCCCAGTGTCATTCCGAGACCCAGTCGGGCATC  ************************************************************  *kdm6bb*_△I5_ I8 AAGTGCATCTCCGACACCCCGTGCTGGTTCAAAATCTAGGCTTTCAGACCTCAAGTACAG  *kdm6bb*_△I5_△I8 AAGTGCATCTCCGACACCCCGTGCTGGTTCAAAATCTAGGCTTTCAGACCTCAAGTACAG  *kdm6bb*_ I5_△I8 AAGTGCATCTCCGACACCCCGTGCTGGTTCAAAATCTAGGCTTTCAGACCTCAAGTACAG  ************************************************************  *kdm6bb*_△I5_ I8 GAAGACCTCACAGCTCCTCTGCTCACGGCAGTCTCCCCACGATAGATCAAGAGTGGACAA  *kdm6bb*_△I5_△I8 GAAGACCTCACAGCTCCTCTGCTCACGGCAGTCTCCCCACGATAGATCAAGAGTGGACAA  *kdm6bb*_ I5_△I8 GAAGACCTCACAGCTCCTCTGCTCACGGCAGTCTCCCCACGATAGATCAAGAGTGGACAA  ************************************************************  *kdm6bb*_△I5_ I8 GCCTGAGAGGGAGTTAGAAGATAAAAAGTCTGAATCCAAACAAAAGTATAAGCTGCTGAA  *kdm6bb*_△I5_△I8 GCCTGAGAGGGAGTTAGAAGATAAAAAGTCTGAATCCAAACAAAAGTATAAGCTGCTGAA  *kdm6bb*_ I5_△I8 GCCTGAGAGGGAGTTAGAAGATAAAAAGTCTGAATCCAAACAAAAGTATAAGCTGCTGAA  ************************************************************  *kdm6bb*_△I5_ I8 AAAAGAGAAGAAGGGTGACGTTCTGAAGATGGGCAGAAAGGGTGAGGACAGAAAAAGGCG  *kdm6bb*_△I5_△I8 AAAAGAGAAGAAGGGTGACGTTCTGAAGATGGGCAGAAAGGGTGAGGACAGAAAAAGGCG  *kdm6bb*_ I5_△I8 AAAAGAGAAGAAGGGTGACGTTCTGAAGATGGGCAGAAAGGGTGAGGACAGAAAAAGGCG  ************************************************************  *kdm6bb*_△I5_ I8 GAAGAAAAAAGAGGAGAAAAGGTTGGCCGAGAAGAAAAAGAAACGCGATAAAGCATCTAA  *kdm6bb*_△I5_△I8 GAAGAAAAAAGAGGAGAAAAGGTTGGCCGAGAAGAAAAAGAAACGCGATAAAGCATCTAA  *kdm6bb*_ I5_△I8 GAAGAAAAAAGAGGAGAAAAGGTTGGCCGAGAAGAAAAAGAAACGCGATAAAGCATCTAA  ************************************************************  *kdm6bb*_△I5_ I8 GAAGGAACGAAAATTAGCCCTCAAAATGAAAGTTACAGAACAGGAAATGTTTTCTTCCGT  *kdm6bb*_△I5_△I8 GAAGGAACGAAAATTAGCCCTCAAAATGAAAGTTACAGAACAGGAAATGTTTTCTTCCGT  *kdm6bb*_ I5_△I8 GAAGGAACGAAAATTAGCCCTCAAAATGAAAGTTACAGAACAGGAAATGTTTTCTTCCGT  ************************************************************  *kdm6bb*_△I5_ I8 CTCTGCTTCCACCTTTCTAAGTGAGGCCAAGATAAGTAAAATGGAGACTTTGAACGGAGA  *kdm6bb*_△I5_△I8 CTCTGCTTCCACCTTTCTAAGTGAGGCCAAGATAAGTAAAATGGAGACTTTGAACGGAGA  *kdm6bb*_ I5_△I8 CTCTGCTTCCACCTTTCTAAGTGAGGCCAAGATAAGTAAAATGGAGACTTTGAACGGAGA  ************************************************************  *kdm6bb*_△I5_ I8 GAATCAGGCCCAGTCACCACCCAAACACAAGCACAGGGACAGGAGTGAAAAAGGAGAGAA  *kdm6bb*_△I5_△I8 GAATCAGGCCCAGTCACCACCCAAACACAAGCACAGGGACAGGAGTGAAAAAGGAGAGAA  *kdm6bb*_ I5_△I8 GAATCAGGCCCAGTCACCACCCAAACACAAGCACAGGGACAGGAGTGAAAAAGGAGAGAA  ************************************************************  *kdm6bb*_△I5_ I8 GACTACGCATAGGTCATCCACAAATACCCTTCATCCCAGGCCCACTTTACAGCAGTCATC  *kdm6bb*_△I5_△I8 GACTACGCATAGGTCATCCACAAATACCCTTCATCCCAGGCCCACTTTACAGCAGTCATC  *kdm6bb*_ I5_△I8 GACTACGCATAGGTCATCCACAAATACCCTTCATCCCAGGCCCACTTTACAGCAGTCATC  ************************************************************  *kdm6bb*_△I5_ I8 CTCAAAGTCTGAAAATCACAAAATGCTCAAAAAGAGCAGCAGTGCCCCAGAAACCCCTCT  *kdm6bb*_△I5_△I8 CTCAAAGTCTGAAAATCACAAAATGCTCAAAAAGAGCAGCAGTGCCCCAGAAACCCCTCT  *kdm6bb*_ I5_△I8 CTCAAAGTCTGAAAATCACAAAATGCTCAAAAAGAGCAGCAGTGCCCCAGAAACCCCTCT  ************************************************************  *kdm6bb*_△I5_ I8 TCCTAAGAAAACCAGCCACCAGGCCAGCAAGCGTTCCACCATCGTCTCAACCCAACTGGA  *kdm6bb*_△I5_△I8 TCCTAAGAAAACCAGCCACCAGGCCAGCAAGCGTTCCACCATCGTCTCAACCCAACTGGA  *kdm6bb*_ I5_△I8 TCCTAAGAAAACCAGCCACCAGGCCAGCAAGCGTTCCACCATCGTCTCAACCCAACTGGA  ************************************************************  *kdm6bb*_△I5_ I8 AGGTGAGCCAAAAGCAAAGCCAGATGACACACTCCCTTCCCTCTTATTTAAAGCCTTAGC  *kdm6bb*_△I5_△I8 AGGTGAGCCAAAAGCAAAGCCAGATGACACACTCCCTTCCCTCTTATTTAAAGCCTTAGC  *kdm6bb*_ I5_△I8 AGGTGAGCCAAAAGCAAAGCCAGATGACACACTCCCTTCCCTCTTATTTAAAGCCTTAGC  ************************************************************  *kdm6bb*_△I5_ I8 GCCTCTCAGTACAGTGTGCTCAGCTGGCTTAGAGCAGCAAGGCGGGCAGGGAGGAGTTCT  *kdm6bb*_△I5_△I8 GCCTCTCAGTACAGTGTGCTCAGCTGGCTTAGAGCAGCAAGGCGGGCAGGGAGGAGTTCT  *kdm6bb*_ I5_△I8 GCCTCTCAGTACAGTGTGCTCAGCTGGCTTAGAGCAGCAAGGCGGGCAGGGAGGAGTTCT  ************************************************************  *kdm6bb*_△I5_ I8 CAACGCTCCAGACCTTCAGCCCGTGGCTGTGATGGGAAATTTAAGGGAAACGGGAGACAA  *kdm6bb*_△I5_△I8 CAACGCTCCAGACCTTCAGCCCGTGGCTGTGATGGGAAATTTAAGGGAAACGGGAGACAA  *kdm6bb*_ I5_△I8 CAACGCTCCAGACCTTCAGCCCGTGGCTGTGATGGGAAATTTAAGGGAAACGGGAGACAA  ************************************************************  *kdm6bb*_△I5_ I8 CCTTGCCAACACACCTCCTGTGCTTAGCTGGCAGGGATCTCCAGTATCAGATCTTGGAGA  *kdm6bb*_△I5_△I8 CCTTGCCAACACACCTCCTGTGCTTAGCTGGCAGGGATCTCCAGTATCAGATCTTGGAGA  *kdm6bb*_ I5_△I8 CCTTGCCAACACACCTCCTGTGCTTAGCTGGCAGGGATCTCCAGTATCAGATCTTGGAGA  ************************************************************  *kdm6bb*_△I5_ I8 TGATGAGGAGGAGCTAGAGAAAGGTGTACTAAGCAGACCCGTCCTCCAGCTCAGCCCCAC  *kdm6bb*_△I5_△I8 TGATGAGGAGGAGCTAGAGAAAGGTGTACTAAGCAGACCCGTCCTCCAGCTCAGCCCCAC  *kdm6bb*_ I5_△I8 TGATGAGGAGGAGCTAGAGAAAGGTGTACTAAGCAGACCCGTCCTCCAGCTCAGCCCCAC  ************************************************************  *kdm6bb*_△I5_ I8 TCAATGTCTTTCACCCCCTCCTGTAGAGAGTGAAAGCATTGATAATATGAATGCAGAAGA  *kdm6bb*_△I5_△I8 TCAATGTCTTTCACCCCCTCCTGTAGAGAGTGAAAGCATTGATAATATGAATGCAGAAGA  *kdm6bb*_ I5_△I8 TCAATGTCTTTCACCCCCTCCTGTAGAGAGTGAAAGCATTGATAATATGAATGCAGAAGA  ************************************************************  *kdm6bb*_△I5_ I8 TTATTCCCACAGCAGCATGTCTGAATTCTCCGATCTACCTTGTTCAATTGAACAAGTTTC  *kdm6bb*_△I5_△I8 TTATTCCCACAGCAGCATGTCTGAATTCTCCGATCTACCTTGTTCAATTGAACAAGTTTC  *kdm6bb*_ I5_△I8 TTATTCCCACAGCAGCATGTCTGAATTCTCCGATCTACCTTGTTCAATTGAACAAGTTTC  ************************************************************  *kdm6bb*_△I5_ I8 GGAGGAAGAAAAGGAAGTGATGATGGACAGCAGAGGGAAGCTGTCTGGCTCTCCCTTTCA  *kdm6bb*_△I5_△I8 GGAGGAAGAAAAGGAAGTGATGATGGACAGCAGAGGGAAGCTGTCTGGCTCTCCCTTTCA  *kdm6bb*_ I5_△I8 GGAGGAAGAAAAGGAAGTGATGATGGACAGCAGAGGGAAGCTGTCTGGCTCTCCCTTTCA  ************************************************************  *kdm6bb*_△I5_ I8 TGAGTTTCATAACGACGACACAGGTTTTGATGATGTCTTTGAGAGCCTGGCCACCTTGCT  *kdm6bb*_△I5_△I8 TGAGTTTCATAACGACGACACAGGTTTTGATGATGTCTTTGAGAGCCTGGCCACCTTGCT  *kdm6bb*_ I5_△I8 TGAGTTTCATAACGACGACACAGGTTTTGATGATGTCTTTGAGAGCCTGGCCACCTTGCT  ************************************************************  *kdm6bb*_△I5_ I8 TGCCAGCCAGAGGGTCGCGTGCCGAGGTGGTCCATTCGGCGGTCCTCCTGCTAGTGATGC  *kdm6bb*_△I5_△I8 TGCCAGCCAGAGGGTCGCGTGCCGAGGTGGTCCATTCGGCGGTCCTCCTGCTAGTGATGC  *kdm6bb*_ I5_△I8 TGCCAGCCAGAGGGTCGCGTGCCGAGGTGGTCCATTCGGCGGTCCTCCTGCTAGTGATGC  ************************************************************  *kdm6bb*_△I5_ I8 AAATGGAGTAAAGTACTCTTGCTCTCTTGAACTGGGCCCAGATATCCACTGCCCCGAGAA  *kdm6bb*_△I5_△I8 AAATGGAGTAAAGTACTCTTGCTCTCTTGAACTGGGCCCAGATATCCACTGCCCCGAGAA  *kdm6bb*_ I5_△I8 AAATGGAGTAAAGTACTCTTGCTCTCTTGAACTGGGCCCAGATATCCACTGCCCCGAGAA  ************************************************************  *kdm6bb*_△I5_ I8 TCAGGACTTCTTCCCTACATCATATCCCACAGGATCCTCCGAACCCAGTAATCAATCACC  *kdm6bb*_△I5_△I8 TCAGGACTTCTTCCCTACATCATATCCCACAGGATCCTCCGAACCCAGTAATCAATCACC  *kdm6bb*_ I5_△I8 TCAGGACTTCTTCCCTACATCATATCCCACAGGATCCTCCGAACCCAGTAATCAATCACC  ************************************************************  *kdm6bb*_△I5_ I8 AGTTCACATTACCACAGACACACTTGAAGAATTCCACAGTCTGGCAGATGTGAGTAAGCC  *kdm6bb*_△I5_△I8 AGTTCACATTACCACAGACACACTTGAAGAATTCCACAGTCTGGCAGATGTGAGTAAGCC  *kdm6bb*_ I5_△I8 AGTTCACATTACCACAGACACACTTGAAGAATTCCACAGTCTGGCAGATGTGAGTAAGCC  ************************************************************  *kdm6bb*_△I5_ I8 AGCCACCAGCTCAATGGTGCAGGAAAAAGAGGAGGAAAATGAGAGTGATGTACAAGGTGC  *kdm6bb*_△I5_△I8 AGCCACCAGCTCAATGGTGCAGGAAAAAGAGGAGGAAAATGAGAGTGATGTACAAGGTGC  *kdm6bb*_ I5_△I8 AGCCACCAGCTCAATGGTGCAGGAAAAAGAGGAGGAAAATGAGAGTGATGTACAAGGTGC  ************************************************************  *kdm6bb*_△I5_ I8 GATGAAACAAGATGGCAAGGATGCAGCAATCACAATGGAGAGGAAGGAGACTCTTTTGGA  *kdm6bb*_△I5_△I8 GATGAAACAAGATGGCAAGGATGCAGCAATCACAATGGAGAGGAAGGAGACTCTTTTGGA  *kdm6bb*_ I5_△I8 GATGAAACAAGATGGCAAGGATGCAGCAATCACAATGGAGAGGAAGGAGACTCTTTTGGA  ************************************************************  *kdm6bb*_△I5_ I8 TGGGTCATTGAGTGCAGAGCTGACTCTGACCACAACACATACAGTAATGAAGATGGTAAA  *kdm6bb*_△I5_△I8 TGGGTCATTGAGTGCAGAGCTGACTCTGACCACAACACATACAGTAATGAAGATGGTAAA  *kdm6bb*_ I5_△I8 TGGGTCATTGAGTGCAGAGCTGACTCTGACCACAACACATACAGTAATGAAGATGGTAAA  ************************************************************  *kdm6bb*_△I5_ I8 GAAAGCTCCCTCTACCAAGGAAAAAAGTGGAAAGGGCACAAAGACAGAACGTGCAGGCAA  *kdm6bb*_△I5_△I8 GAAAGCTCCCTCTACCAAGGAAAAAAGTGGAAAGGGCACAAAGACAGAACGTGCAGGCAA  *kdm6bb*_ I5_△I8 GAAAGCTCCCTCTACCAAGGAAAAAAGTGGAAAGGGCACAAAGACAGAACGTGCAGGCAA  ************************************************************  *kdm6bb*_△I5_ I8 AGAGAAAAAGAGAAAACAGAAGGTAAAGGATGGGAAAGGAGAGGGAGCGATCAAGATTAA  *kdm6bb*_△I5_△I8 AGAGAAAAAGAGAAAACAGAAGGTAAAGGATGGGAAAGGAGAGGGAGCGATCAAGATTAA  *kdm6bb*_ I5_△I8 AGAGAAAAAGAGAAAACAGAAGGTAAAGGATGGGAAAGGAGAGGGAGCGATCAAGATTAA  ************************************************************  *kdm6bb*_△I5_ I8 GATAAAGAGAGGAGAAAATAAGGTCATCAGCTGTAAAATTAAAACAAGTAGAGTACCTGA  *kdm6bb*_△I5_△I8 GATAAAGAGAGGAGAAAATAAGGTCATCAGCTGTAAAATTAAAACAAGTAGAGTACCTGA  *kdm6bb*_ I5_△I8 GATAAAGAGAGGAGAAAATAAGGTCATCAGCTGTAAAATTAAAACAAGTAGAGTACCTGA  ************************************************************  *kdm6bb*_△I5_ I8 TGTTAAAGACAGCGACACTTCATCTTCAGTGCCAGTTATTTCATACTCAGCCAGACCGGT  *kdm6bb*_△I5_△I8 TGTTAAAGACAGCGACACTTCATCTTCAGTGCCAGTTATTTCATACTCAGCCAGACCGGT  *kdm6bb*_ I5_△I8 TGTTAAAGACAGCGACACTTCATCTTCAGTGCCAGTTATTTCATACTCAGCCAGACCGGT  ************************************************************  *kdm6bb*_△I5_ I8 CAAAGACAGCATGAAGGGCCAGACTCCTCAGGAAAATCAAACCCCAAATGGCAAAAATAT  *kdm6bb*_△I5_△I8 CAAAGACAGCATGAAGGGCCAGACTCCTCAGGAAAATCAAACCCCAAATGGCAAAAATAT  *kdm6bb*_ I5_△I8 CAAAGACAGCATGAAGGGCCAGACTCCTCAGGAAAATCAAACCCCAAATGGCAAAAATAT  ************************************************************  *kdm6bb*_△I5_ I8 CGAAAGACAGAAAATGGACACTGGGAATTCAAATGCAACAACAGATGAGAAGAAAGGGTT  *kdm6bb*_△I5_△I8 CGAAAGACAGAAAATGGACACTGGGAATTCAAATGCAACAACAGATGAGAAGAAAGGGTT  *kdm6bb*_ I5_△I8 CGAAAGACAGAAAATGGACACTGGGAATTCAAATGCAACAACAGATGAGAAGAAAGGGTT  ************************************************************  *kdm6bb*_△I5_ I8 AGATAATGAGACTGCCTGTGTAGTCGGAATTACAGACATCACCACCTCAAATTCAGCATC  *kdm6bb*_△I5_△I8 AGATAATGAGACTGCCTGTGTAGTCGGAATTACAGACATCACCACCTCAAATTCAGCATC  *kdm6bb*_ I5_△I8 AGATAATGAGACTGCCTGTGTAGTCGGAATTACAGACATCACCACCTCAAATTCAGCATC  ************************************************************  *kdm6bb*_△I5_ I8 AGCTGTATCAACAAGTCCATCCAAATTAGATCGTCCTACACCAGCAAGTAAAGTGGACCC  *kdm6bb*_△I5_△I8 AGCTGTATCAACAAGTCCATCCAAATTAGATCGTCCTACACCAGCAAGTAAAGTGGACCC  *kdm6bb*_ I5_△I8 AGCTGTATCAACAAGTCCATCCAAATTAGATCGTCCTACACCAGCAAGTAAAGTGGACCC  ************************************************************  *kdm6bb*_△I5_ I8 ACTGAAACTGAAAGCGCTGTCCATGGGCTTGTCTAAGGAGCTGAAGATCCTCTTGGTTAA  *kdm6bb*_△I5_△I8 ACTGAAACTGAAAGCGCTGTCCATGGGCTTGTCTAAGGAGCTGAAGATCCTCTTGGTTAA  *kdm6bb*_ I5_△I8 ACTGAAACTGAAAGCGCTGTCCATGGGCTTGTCTAAGGAGCTGAAGATCCTCTTGGTTAA  ************************************************************  *kdm6bb*_△I5_ I8 AGTGGAGAGCGGCGGAAGGCAGACATTCAACATATCAGAGGTGCAGGAGCAACGAATCCC  *kdm6bb*_△I5_△I8 AGTGGAGAGCGGCGGAAGGCAGACATTCAACATATCAGAGGTGCAGGAGCAACGAATCCC  *kdm6bb*_ I5_△I8 AGTGGAGAGCGGCGGAAGGCAGACATTCAACATATCAGAGGTGCAGGAGCAACGAATCCC  ************************************************************  *kdm6bb*_△I5_ I8 AATCCCACTTTCCAAGATCAGCATCACAAATACAGCCGCTGATGTGATCGGAGCATGCAG  *kdm6bb*_△I5_△I8 AATCCCACTTTCCAAGATCAGCATCACAAATACAGCCGCTGATGTGATCGGAGCATGCAG  *kdm6bb*_ I5_△I8 AATCCCACTTTCCAAGATCAGCATCACAAATACAGCCGCTGATGTGATCGGAGCATGCAG  ************************************************************  *kdm6bb*_△I5_ I8 GGGGGCAAGTGTGAAAGGGAAATTCAAGGAGTCGTACTTGCCTCCCGCATTTTCGGTTAA  *kdm6bb*_△I5_△I8 GGGGGCAAGTGTGAAAGGGAAATTCAAGGAGTCGTACTTGCCTCCCGCATTTTCGGTTAA  *kdm6bb*_ I5_△I8 GGGGGCAAGTGTGAAAGGGAAATTCAAGGAGTCGTACTTGCCTCCCGCATTTTCGGTTAA  ************************************************************  *kdm6bb*_△I5_ I8 ACCCAACATTGCAGCTGAGACCCCCATTCCTCGCGATAAGCTGAATCCTCCTACACCAAG  *kdm6bb*_△I5_△I8 ACCCAACATTGCAGCTGAGACCCCCATTCCTCGCGATAAGCTGAATCCTCCTACACCAAG  *kdm6bb*_ I5_△I8 ACCCAACATTGCAGCTGAGACCCCCATTCCTCGCGATAAGCTGAATCCTCCTACACCAAG  ************************************************************  *kdm6bb*_△I5_ I8 CATCTATTTGGAGAGCAAGAGGGATGCCTTTTCTCCGGTTCTGCTTCAGTTCTGCACTGA  *kdm6bb*_△I5_△I8 CATCTATTTGGAGAGCAAGAGGGATGCCTTTTCTCCGGTTCTGCTTCAGTTCTGCACTGA  *kdm6bb*_ I5_△I8 CATCTATTTGGAGAGCAAGAGGGATGCCTTTTCTCCGGTTCTGCTTCAGTTCTGCACTGA  ************************************************************  *kdm6bb*_△I5_ I8 TCCCAAAAACGCTGTTACGGTCATCAGAGGCCTCGCTGGCTCCCTCCGCCTTAATCTTGG  *kdm6bb*_△I5_△I8 TCCCAAAAACGCTGTTACGGTCATCAGAGGCCTCGCTGGCTCCCTCCGCCTTAATCTTGG  *kdm6bb*_ I5_△I8 TCCCAAAAACGCTGTTACGGTCATCAGAGGCCTCGCTGGCTCCCTCCGCCTTAATCTTGG  ************************************************************  *kdm6bb*_△I5_ I8 TCTGTTCTCAACCAAATCTCTGGTGGAGGCCAATTCGGACCATGCAGTGGAAGTGAGGAC  *kdm6bb*_△I5_△I8 TCTGTTCTCAACCAAATCTCTGGTGGAGGCCAATTCGGACCATGCAGTGGAAGTGAGGAC  *kdm6bb*_ I5_△I8 TCTGTTCTCAACCAAATCTCTGGTGGAGGCCAATTCGGACCATGCAGTGGAAGTGAGGAC  ************************************************************  *kdm6bb*_△I5_ I8 TCAGGTTCAGCAGCCTGCTGATGAGAACTGGGATCCAAGTGGTGCGACTCAGACGTGGCC  *kdm6bb*_△I5_△I8 TCAGGTTCAGCAGCCTGCTGATGAGAACTGGGATCCAAGTGGTGCGACTCAGACGTGGCC  *kdm6bb*_ I5_△I8 TCAGGTTCAGCAGCCTGCTGATGAGAACTGGGATCCAAGTGGTGCGACTCAGACGTGGCC  ************************************************************  *kdm6bb*_△I5_ I8 CTGCGAGAGCAGCCGCTCACACACCACCATTGCCAAATATGCCCAGTACCAGGCCTCCAC  *kdm6bb*_△I5_△I8 CTGCGAGAGCAGCCGCTCACACACCACCATTGCCAAATATGCCCAGTACCAGGCCTCCAC  *kdm6bb*_ I5_△I8 CTGCGAGAGCAGCCGCTCACACACCACCATTGCCAAATATGCCCAGTACCAGGCCTCCAC  ************************************************************  *kdm6bb*_△I5_ I8 CTTTCAGGAGAGCCTGGAGGAGGAGAAGGAGAGTGAGAATGAGGAAGACGAGGAACAGAC  *kdm6bb*_△I5_△I8 CTTTCAGGAGAGCCTGGAGGAGGAGAAGGAGAGTGAGAATGAGGAAGACGAGGAACAGAC  *kdm6bb*_ I5_△I8 CTTTCAGGAGAGCCTGGAGGAGGAGAAGGAGAGTGAGAATGAGGAAGACGAGGAACAGAC  ************************************************************  *kdm6bb*_△I5_ I8 TCCAAACCAATCGGCGTCCACAAAAGCCGCTCTGACATTATCCAACAGTAAAGGCAATCA  *kdm6bb*_△I5_△I8 TCCAAACCAATCGGCGTCCACAAAAGCCGCTCTGACATTATCCAACAGTAAAGGCAATCA  *kdm6bb*_ I5_△I8 TCCAAACCAATCGGCGTCCACAAAAGCCGCTCTGACATTATCCAACAGTAAAGGCAATCA  ************************************************************  *kdm6bb*_△I5_ I8 TACCTCAAAAGCCAGTTCTGCGTCCATCTTGAGCAAAGCTCAGCCTCCTAATGCCAACAG  *kdm6bb*_△I5_△I8 TACCTCAAAAGCCAGTTCTGCGTCCATCTTGAGCAAAGCTCAGCCTCCTAATGCCAACAG  *kdm6bb*_ I5_△I8 TACCTCAAAAGCCAGTTCTGCGTCCATCTTGAGCAAAGCTCAGCCTCCTAATGCCAACAG  ************************************************************  *kdm6bb*_△I5_ I8 CTCTCTGAGCTCAGAGCAAAAACCTGTTGGAAAGATCATTAAATTCGGGACCAACATAGA  *kdm6bb*_△I5_△I8 CTCTCTGAGCTCAGAGCAAAAACCTGTTGGAAAGATCATTAAATTCGGGACCAACATAGA  *kdm6bb*_ I5_△I8 CTCTCTGAGCTCAGAGCAAAAACCTGTTGGAAAGATCATTAAATTCGGGACCAACATAGA  ************************************************************  *kdm6bb*_△I5_ I8 TCTGTCTGATCCTAAAAGGTGGAAGCCCCAGCTGCAGGAGCTGCTGAAGCTGCCAGCTTT  *kdm6bb*_△I5_△I8 TCTGTCTGATCCTAAAAGGTGGAAGCCCCAGCTGCAGGAGCTGCTGAAGCTGCCAGCTTT  *kdm6bb*_ I5_△I8 TCTGTCTGATCCTAAAAGGTGGAAGCCCCAGCTGCAGGAGCTGCTGAAGCTGCCAGCTTT  ************************************************************  *kdm6bb*_△I5_ I8 CATGCGAGTGGAATCCAGCAACAACATGCTGAGTCACGTCGGTCACACCATCCTGGGCAT  *kdm6bb*_△I5_△I8 CATGCGAGTGGAATCCAGCAACAACATGCTGAGTCACGTCGGTCACACCATCCTGGGCAT  *kdm6bb*_ I5_△I8 CATGCGAGTGGAATCCAGCAACAACATGCTGAGTCACGTCGGTCACACCATCCTGGGCAT  ************************************************************  *kdm6bb*_△I5_ I8 GAACACTGTCCAGCTTTACATGAAGGTGCCAGGCAGCCGTACGCCAGGTCATCAAGAGAA  *kdm6bb*_△I5_△I8 GAACACTGTCCAGCTTTACATGAAGGTGCCAGGCAGCCGTACGCCAGGTCATCAAGAGAA  *kdm6bb*_ I5_△I8 GAACACTGTCCAGCTTTACATGAAGGTGCCAGGCAGCCGTACGCCAGGTCATCAAGAGAA  ************************************************************  *kdm6bb*_△I5_ I8 CAACAATTTCTGCTCAGTTAACATCAACATCGGGCCCGGTGACTGTGAGTGGTTCGCAGT  *kdm6bb*_△I5_△I8 CAACAATTTCTGCTCAGTTAACATCAACATCGGGCCCGGTGACTGTGAGTGGTTCGCAGT  *kdm6bb*_ I5_△I8 CAACAATTTCTGCTCAGTTAACATCAACATCGGGCCCGGTGACTGTGAGTGGTTCGCAGT  ************************************************************  *kdm6bb*_△I5_ I8 CCATGAACACTACTGGGAACTTATTAACAATTTATGTGAAAAGCATGGAGTAGACTACCT  *kdm6bb*_△I5_△I8 CCATGAACACTACTGGGAACTTATTAACAATTTATGTGAAAAGCATGGAGTAGACTACCT  *kdm6bb*_ I5_△I8 CCATGAACACTACTGGGAACTTATTAACAATTTATGTGAAAAGCATGGAGTAGACTACCT  ************************************************************  *kdm6bb*_△I5_ I8 TACAGGGTCCTGGTGGCCAGTTCTAGAGGATCTCTACAGTTCCAACATCCCTGTGTACCG  *kdm6bb*_△I5_△I8 TACAGGGTCCTGGTGGCCAGTTCTAGAGGATCTCTACAGTTCCAACATCCCTGTGTACCG  *kdm6bb*_ I5_△I8 TACAGGGTCCTGGTGGCCAGTTCTAGAGGATCTCTACAGTTCCAACATCCCTGTGTACCG  ************************************************************  *kdm6bb*_△I5_ I8 CTTCATCCAGAGGCCAGGCGACCTGGTGTGGATTAATGCAGGAACTGTGCACTGGGTCCA  *kdm6bb*_△I5_△I8 CTTCATCCAGAGGCCAGGCGACCTGGTGTGGATTAATGCAGGAACTGTGCACTGGGTCCA  *kdm6bb*_ I5_△I8 CTTCATCCAGAGGCCAGGCGACCTGGTGTGGATTAATGCAGGAACTGTGCACTGGGTCCA  ************************************************************  *kdm6bb*_△I5_ I8 AGCTGTGGGCTGGTGCAACAACATTGCCTGGAATGTGGGACCGCTCAACTCATACCAATA  *kdm6bb*_△I5_△I8 AGCTGTGGGCTGGTGCAACAACATTGCCTGGAATGTGGGACCGCTCAACTCATACCAATA  *kdm6bb*_ I5_△I8 AGCTGTGGGCTGGTGCAACAACATTGCCTGGAATGTGGGACCGCTCAACTCATACCAATA  ************************************************************  *kdm6bb*_△I5_ I8 TCAACTCGCCCTGGAGCGCTTTGAGTGGAACGAGGTGAAGAAGGTTAAGTCAATCGTTCC  *kdm6bb*_△I5_△I8 TCAACTCGCCCTGGAGCGCTTTGAGTGGAACGAGGTGAAGAAGGTTAAGTCAATCGTTCC  *kdm6bb*_ I5_△I8 TCAACTCGCCCTGGAGCGCTTTGAGTGGAACGAGGTGAAGAAGGTTAAGTCAATCGTTCC  ************************************************************  *kdm6bb*_△I5_ I8 CATGATCCACGTTTCCTGGAATGTGGCTCGCACCATTAAAATCACAGATCAGGATACCTT  *kdm6bb*_△I5_△I8 CATGATCCACGTTTCCTGGAATGTGGCTCGCACCATTAAAATCACAGATCAGGATACCTT  *kdm6bb*_ I5_△I8 CATGATCCACGTTTCCTGGAATGTGGCTCGCACCATTAAAATCACAGATCAGGATACCTT  ************************************************************  *kdm6bb*_△I5_ I8 CAAGATGATCAAACACTGCCTGTTGCAGTCCATCAAGCACATCCAGATTCTGAGAGACCA  *kdm6bb*_△I5_△I8 CAAGATGATCAAACACTGCCTGTTGCAGTCCATCAAGCACATCCAGATTCTGAGAGACCA  *kdm6bb*_ I5_△I8 CAAGATGATCAAACACTGCCTGTTGCAGTCCATCAAGCACATCCAGATTCTGAGAGACCA  ************************************************************  *kdm6bb*_△I5_ I8 GCTGGTGGCTGCAGGGAAGAAAATCTGTTACCAGAGCCGCGTGAAAGACGAGCCAGCCTA  *kdm6bb*_△I5_△I8 GCTGGTGGCTGCAGGGAAGAAAATCTGTTACCAGAGCCGCGTGAAAGACGAGCCAGCCTA  *kdm6bb*_ I5_△I8 GCTGGTGGCTGCAGGGAAGAAAATCTGTTACCAGAGCCGCGTGAAAGACGAGCCAGCCTA  ************************************************************  *kdm6bb*_△I5_ I8 CTACTGCAACGAGTGTGATGTGGAGGTGTTTAACTTGCTGTTTGTGACAAGTGAGAACAG  *kdm6bb*_△I5_△I8 CTACTGCAACGAGTGTGATGTGGAGGTGTTTAACTTGCTGTTTGTGACAAGTGAGAACAG  *kdm6bb*_ I5_△I8 CTACTGCAACGAGTGTGATGTGGAGGTGTTTAACTTGCTGTTTGTGACAAGTGAGAACAG  ************************************************************  *kdm6bb*_△I5_ I8 TAGTAAGAAGACCTACGTGGTGCACTGTGAGGACTGTGCCAGAGCTAAGAACCCGTCGCT  *kdm6bb*_△I5_△I8 TAGTAAGAAGACCTACGTGGTGCACTGTGAGGACTGTGCCAGAGCTAAGAACCCGTCGCT  *kdm6bb*_ I5_△I8 TAGTAAGAAGACCTACGTGGTGCACTGTGAGGACTGTGCCAGAGCTAAGAACCCGTCGCT  ************************************************************  *kdm6bb*_△I5_ I8 GACAGGAGTAGTGGTGCTGGAACAGTATCGGATAGAGGAGCTGATGAAAATCTATGACAG  *kdm6bb*_△I5_△I8 GACAGGAGTAGTGGTGCTGGAACAGTATCGGATAGAGGAGCTGATGAAAATCTATGACAG  *kdm6bb*_ I5_△I8 GACAGGAGTAGTGGTGCTGGAACAGTATCGGATAGAGGAGCTGATGAAAATCTATGACAG  ************************************************************  *kdm6bb*_△I5_ I8 TTTTGTGCTGACTCCAACTCCCTTCTCAAAGTGA  *kdm6bb*_△I5_△I8 TTTTGTGCTGACTCCAACTCCCTTCTCAAAGTGA  *kdm6bb*_ I5_△I8 TTTTGTGCTGACTCCAACTCCCTTCTCAAAGTGA  ********************************** |
